# Neural dynamics of shifting attention between perception and working-memory contents

**DOI:** 10.1073/pnas.2406061121

**Published:** 2024-11-13

**Authors:** Daniela Gresch, Sage E. P. Boettcher, Chetan Gohil, Freek van Ede, Anna C. Nobre

**Affiliations:** ^a^Department of Experimental Psychology, University of Oxford, Oxford OX2 6GG, United Kingdom; ^b^Oxford Centre for Human Brain Activity, Wellcome Centre for Integrative Neuroimaging, Department of Psychiatry, University of Oxford, Oxford OX3 7JX, United Kingdom; ^c^Department of Psychology, Yale University, New Haven, CT 06510; ^d^Institute for Brain and Behaviour Amsterdam, Department of Experimental and Applied Psychology, Vrije Universiteit Amsterdam, Amsterdam 1081 HV, The Netherlands; ^e^Wu Tsai Institute, Yale University, New Haven, CT 06510

**Keywords:** external attention, internal attention, MEG, decoding, alpha

## Abstract

In everyday tasks, our focus of attention shifts seamlessly between contents in the sensory environment and internal memory representations. Yet, research has mainly considered external and internal attention in isolation. We used magnetoencephalography to compare the neural dynamics of shifting attention to visual contents within vs. between the external and internal domains. Participants performed a combined perception and working-memory task in which two sequential cues guided attention to upcoming (external) or memorized (internal) sensory information. Critically, the second cue could redirect attention to visual content within the same or alternative domain as the first cue. Multivariate decoding unveiled distinct patterns of human brain activity when shifting attention within vs. between domains. Brain activity distinguishing within- from between-domain shifts was broadly distributed and highly dynamic. Intriguingly, crossing domains did not invoke an additional stage prior to shifting attention. Alpha lateralization, a canonical marker of shifting spatial attention, showed no delay when cues redirected attention to the same vs. alternative domain. Instead, evidence suggested that neural states associated with a given domain linger and influence subsequent shifts of attention within vs. between domains. Our findings provide critical insights into the neural dynamics that govern attentional shifts between perception and working memory.

## Significance

During almost every natural behavior, our attention regularly shifts between sensory and memory contents. Although the systems and mechanisms of attentional control and modulation within the external and internal domains have been heavily studied in isolation, how attention crosses between these domains remains uncharted territory. Our study investigates the brain dynamics associated with shifting attention between contents in the sensory environment and memory representations. By using a combined perception and working-memory task, we were able to isolatebrain activity associated with shifting attention within vs. between the external and internal domains. Our findings reveal early, dynamic, and distributed patterns of activity that distinguish within- from between-domain shifts, offering fascinating initial insights that give rise to further investigations.

Attention can be selectively directed toward sensory information within the external environment or internal representations in memory, phenomena referred to as external and internal attention, respectively (1). During almost every natural behavior, our attention fluctuates seamlessly between these attentional domains, rapidly transitioning focus between sensory and mnemonic contents. Despite playing an integral role in daily cognition, the mechanisms underlying attentional shifts between perceptual and memory contents have been relatively overlooked.

A large body of research has focused on studying isolated shifts of attention within perception (2, 3) or within working memory (4–6). Some investigations have also considered how shifts within each domain compare, emphasizing shared neural systems and dynamics as well as highlighting dissociations (7–15). These studies have typically employed informative pre-cues or retro-cues in their task design to isolate brain activity specifically associated with shifting attention in either domain (4, 8–10, 14, 15).

In contrast, the investigation of between-domain shifts—transitioning attention between perceptual and mnemonic contents—is still at a nascent stage. A limited number of behavioral studies have highlighted higher costs when shifting across different attentional domains compared to shifting within the same domain (16–21). Brain-imaging and electrophysiological studies comparing task phases that rely more heavily on sensory stimuli vs. internal states have indicated that the lateral prefrontal cortex contributes to shifting attention between the external and internal domains (22–24). Complementary lines of research have suggested the hippocampus (25, 26), fronto-insular cortex (27), or retro-splenial cortex (28) may serve as interfaces between perception and memory. However, it is important to note that the task designs of these studies did not specifically contrast attentional shifts within and between domains. Beyond these initial clues, the dynamic neural mechanisms of cross-domain attention shifts remain elusive.

We aimed to expand upon these initial steps by investigating the brain dynamics associated with shifting attention between the perceptual and working-memory domains and by directly comparing these to the dynamics linked to shifting attention within domains. Building on our recent behavioral study (17), we used a combined perception and working-memory task in which informative cues indicated the location of sensory or mnemonic contents for subsequent report. Successive cues necessitated shifts of attention either within or between perception and working memory. We recorded magnetoencephalography (MEG) to track brain activity with high temporal resolution while participants performed the task and used multivariate pattern analysis to compare activity related to attentional shifts of different types. Based on our and other previous findings of performance costs for shifting attention between relative to within domains (16–21), we hypothesized the engagement of distinct processes when shifting attention between domains.

To preview our results, we demonstrate that dissociable patterns of brain activity accompany shifts between perception and working memory as opposed to shifts within either of these domains. These unique patterns of brain activity are broadly distributed, temporally dynamic, and occur without interfering with the timing of spatial attention shifts as indexed by the lateralization of posterior 8 to 12 Hz alpha activity. In addition, we show distinct patterns of brain activity are associated with external and internal attention states themselves. These findings provide important initial clues about the dynamic neural bases that govern shifts of attention between perception and working-memory contents.

## Results

**Fig. 1.**
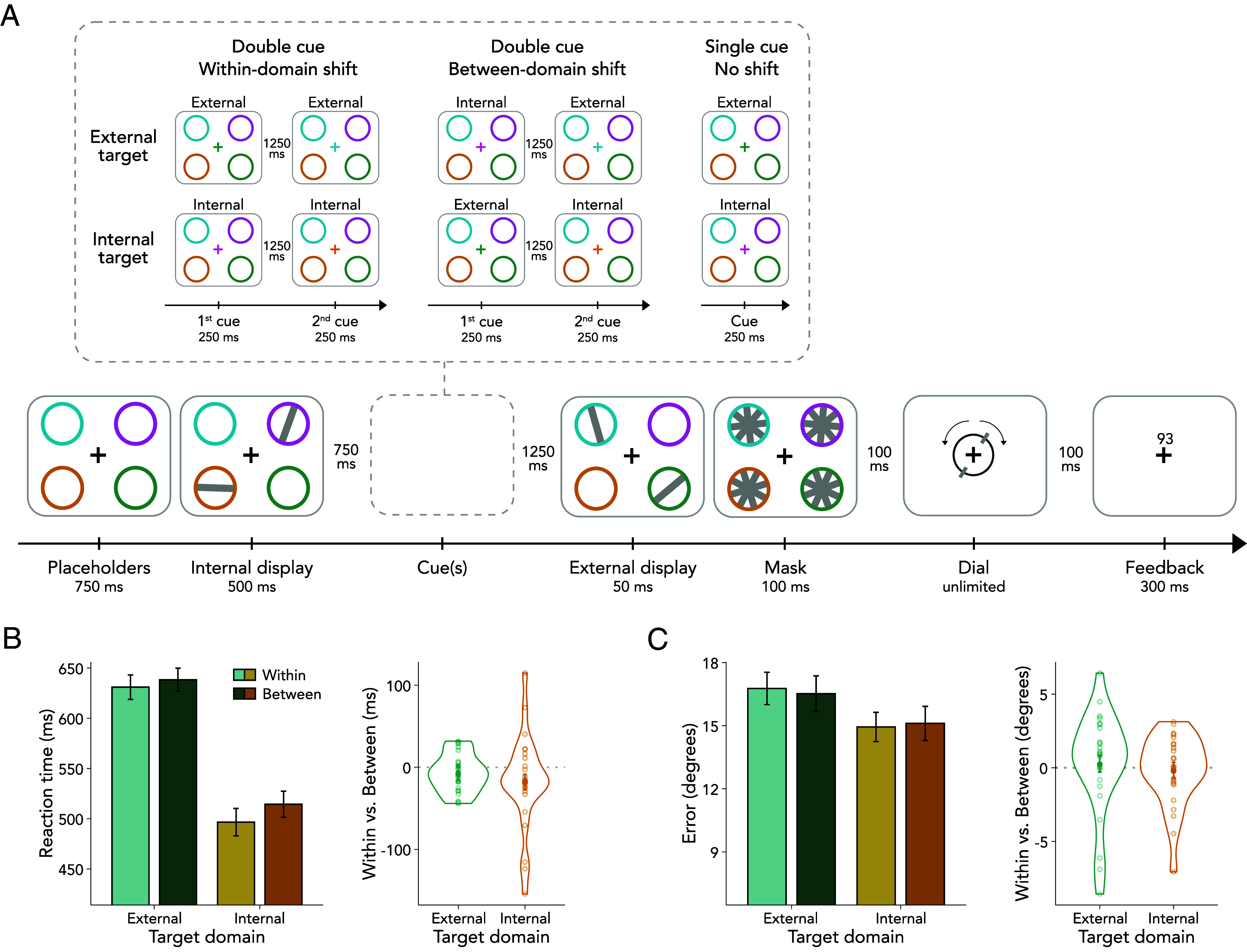
Task schematic and behavioral results. (*A*) Tilted bars were presented across a working-memory array, encoded before the cues (i.e., internal display), and a perceptual array, appearing after the cues (i.e., external display). In double-cue (i.e., shift) trials, two cues appeared between the presentation of the internal and external display. At the time of the second cue, attention needed to be reoriented either to an item of the same domain (i.e., within-domain shift) or to an item in the other domain (i.e., between-domain shift). To ensure participants relied on the first cue, 50% single-cue trials were included in which only one cue was presented, and thus no attentional reallocation was required. (*B*) *Left* panel: Average reaction times (RTs) as a function of target domain and shift type. RTs were faster for internal than external targets and for within- than between-domain shifts. The interaction between target domain × shift type was not significant. Error bars represent SEM. *Right* panel: Shift cost in RTs defined as the difference between shift types (i.e., within minus between) for each target domain. Dots represent individual participants. (*C*) *Left* panel: Same as *Left* panel in (*B*), but for reproduction errors. There were neither significant main nor interaction effects. *Right* panel: Same as *Right* panel in (*B*), but for reproduction errors.

Twenty-five healthy human volunteers performed a combined perception and working-memory task (Fig. 1*A*). Two separate bilateral arrays each containing two randomly oriented bars were presented, with one array occurring before and the other after one or two spatially informative color cues. That is, two bars appeared in an early, internal working-memory array, encoded before the cues, and two bars in a later, external perceptual array, appearing after the cues. At the end of each trial, participants reproduced the orientation of the bar that was last cued. Cues indicating the location of a previously encoded bar guided attention internally (i.e., retro-cues, internal cues), instructing which item in working memory was relevant for reporting. In contrast, cues indicating a previously unoccupied location directed attention externally (i.e., pre-cues, external cues), instructing which item in the upcoming perceptual array would be relevant. Half of the trials were no-shift trials in which only a single cue was presented, and participants reported the initially cued item. These randomly intermixed no-shift trials were included only to ensure that participants relied on the initial cue when performing the task. In the remaining trials, participants were tasked with shifting their attention to another item, which could be either an external or internal item, as indicated by a second cue. These double-cue trials were of central interest. The second cue always pointed to another item in the opposite hemifield but, importantly, equally often indicated that attention needed to be reoriented to an item of the same domain (i.e., within-domain shift) or an item in the other domain (i.e., between-domain shift). This resulted in four possible shift conditions: external-to-external, internal-to-internal, external-to-internal, and internal-to-external. The orthogonal manipulation of the to-be-reported target domain (external vs. internal) and the shift type (within-domain shift vs. between-domain shift) enabled us to investigate these two factors independently. The first cue in single-cue trials and the second cue in double-cue trials were 100% instructive and, thus, always indicated the target for report (*SI Appendix*, *Methods and Materials* for a detailed description).

### Shifting Attention Between Domains Incurs a Behavioral Cost.

Our analysis focused on double-cue trials, as they uniquely enabled us to compare within- and between-domain shifts. Fig. 1*B* depicts the average response-initiation times (RTs) relative to dial onset as a function of the target domain and shift type. We found longer RTs when reorienting attention between domains compared to reorienting within the same domain [*F*_(1,24)_ = 6.466, *P* = 0.018, η^2^_G_ = 0.003]. This between-domain shift cost replicates previous behavioral findings (16–21). Moreover, RTs to internal targets were faster than to external targets [*F*_(1,24)_ = 44.240, *P* < 0.001, η^2^_G_ = 0.216], likely because behavioral responses to the former could be prepared ahead of the response stage (29, 30). The interaction between target domain and shift type was not significant [*F*_(1,24)_ = 1.280, *P* = 0.269, η^2^_G_ < 0.001], suggesting that the effect of shift type did not depend on target domain (i.e., whether participants reported an internal or an external item).

As shown in Fig. 1*C*, reproduction errors were similar in magnitude, yielding no significant main effect [target domain: *F*_(1,24)_ = 0.729, *P* = 0.402, η^2^_G_ = 0.008; shift type: *F*_(1,24)_ = 0.307, *P* = 0.585, η^2^_G_ < 0.001] or interaction [*F*_(1,24)_ = 1.552, *P* = 0.225, η^2^_G_ = 0.002].

### Distinct Patterns of Brain Activity for Within- vs. Between-Domain Shifts.

Our main aim was to investigate whether and when within- and between-domain shifts evoked reliably different neural activity patterns. To this end, we applied a time-resolved cross-validation decoding approach to the broadband sensor-level MEG data collected while participants performed the task. We first trained a Linear Discriminant Analysis (LDA) classifier to distinguish external-to-external and internal-to-internal trials (i.e., within-domain shifts) from external-to-internal and internal-to-external trials (i.e., between-domain shifts) on a timepoint-by-timepoint basis. We used the Area Under the Curve of the Receiver Operating Characteristic Curve (ROC-AUC) as the evaluation metric (31). The orthogonal manipulation of the target domain, shift type, and cued location ensured that the decoding of within- vs. between-domain shifts could be isolated, without being influenced by merely detecting the currently cued attentional domain or cued location.

**Fig. 2.**
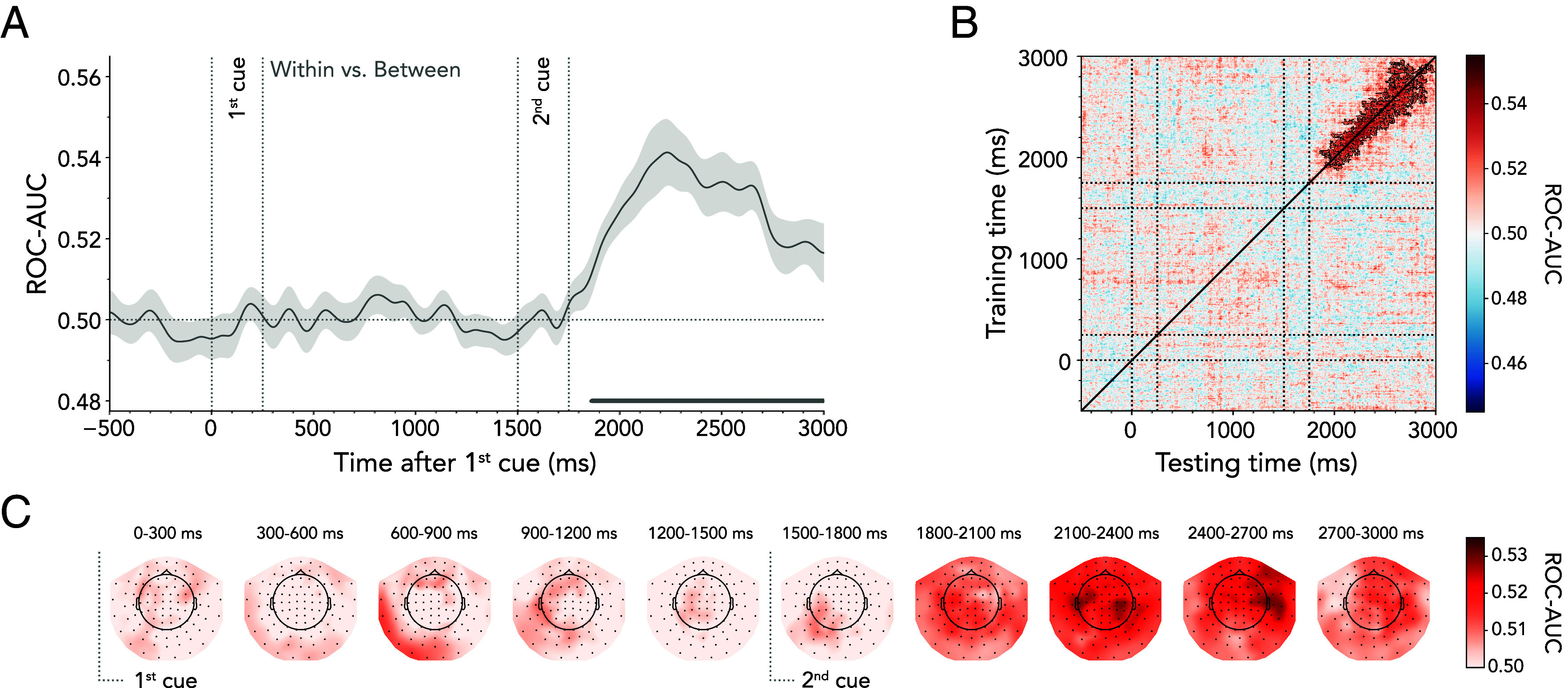
Time-resolved MEG decoding of within- vs. between-domain shifts of attention. (*A*) Average classifier performance for discriminating within- from between-domain shifts plotted over time. Time course shows M ± SEM across participants. Cluster-permutation corrected significant time points are indicated with horizontal lines. (*B*) Temporal-generalization matrix reflecting classifier performance in discriminating the two shift types. The diagonal corresponds to the time course illustrated in (*A*). Black outlines indicate significant decoding clusters. (*C*) The MEG topography averages in 300-ms steps showing which sensors most strongly contribute to the overall decoding performance.

We found reliable decoding of within- vs. between-domain shifts. As expected, no decoding of reorienting attention within vs. between domains was observed until after the second cue (Fig. 2*A*). Within- vs. between-shift decoding reached significance approximately 360 ms after the second-cue onset and persisted until external display presentation (~1,860 to 3,000 ms, cluster *P* < 0.001). Complementary univariate analyses of event-related fields (ERFs) were insufficient to detect differences between shift types (*SI Appendix*, Fig. S1). This suggests that the reported multivariate differences may be driven by the pattern of activity rather than the amplitude of evoked responses.

Since involuntary eye movements may contaminate MEG recordings and lead to decodable nonbrain signals (32, 33), we trained a classifier to distinguish within- vs. between-domain shifts, but this time using only the x- and y-coordinates of the eye-tracking data. We could not decode the shift type based on the eye-tracking signal (*SI Appendix*, Fig. S2*A*). Moreover, we assessed whether there was mutual information between the eye-tracking and the MEG data. *SI Appendix*, Fig. S2*B*, shows the mutual information, with values of zero indicating that the MEG and eye-position signals are independent and larger values indicating a greater relationship between them. There was almost no mutual information between the eye-tracking and the MEG data when decoding within- vs. between-domain shifts. However, two participants were outliers, exhibiting stronger mutual information than the rest (*SI Appendix*, Fig. S2*C*). We therefore repeated the MEG decoding analysis while excluding these participants. This revealed that MEG-based decoding was not driven by participants with strong mutual information (*SI Appendix*, Fig. S2*D*).

To assess whether the pattern of neural activity underlying the classification performance was stable or evolved dynamically over time, we performed a generalization-across-time analysis (34, 35). Significant points off the diagonal would indicate that the classifier, when trained on data from time point A, can generalize to data from time point B. In our case, the evolving pattern of neural activity that distinguished within- from between-domain attention shifts was highly dynamic. The classifier only significantly generalized over brief periods (Fig. 2*B*; 1st cluster *P* < 0.001, 2nd cluster *P* = 0.027). This indicates that although we can consistently distinguish between trials in which participants shifted attention within vs. between domains, the neural code driving this distinction varied moment-to-moment. Our primary decoding analysis included data from all MEG sensor locations as features in the classifier. A searchlight analysis (i.e., iterative decoding for subsets of MEG sensors) was used to characterize the scalp distribution of sensors sensitive to within vs. between domain shifts. Fig. 2*C* shows that the neural patterns distinguishing within- vs. between-domain shifts were broadly distributed.

In an exploratory analysis, we tested whether patterns of brain activity discriminating within- from between-domain shifts impacted later behavioral performance. To this end, we obtained single-trial decoding scores for each participant within the 360 to 1,500 ms interval following the second cue (i.e., the significant cluster interval shown in Fig. 2*A*) and categorized these trial scores into low vs. high decoding performance. *SI Appendix*, Fig. S3 depicts RTs as a function of shift type (within vs. between) and median split (low shift-type decoding vs. high shift-type decoding). Pairwise comparisons revealed a promising trend, showing a significant between-domain shift cost only when comparing trials with high classifier performance [*t*_(24)_ = 2.678, *P* = 0.013, *d* = 0.536]. In contrast, there was no significant difference between shift types when considering trials with low classifier performance [*t*_(24)_ = 0.473, *P* = 0.641, *d* = 0.095]. However, the direct comparison of the shift cost between low and high shift-type decoding did not produce significant results [*t*_(24)_ = 1.769, *P* = 0.090, *d* = 0.354]. Thus, while the results tentatively suggest that the ability to decode within- vs. between-domain shifts may impact later behavioral performance, these findings are not yet conclusive.

Together, our data show distinct patterns of brain activity associated with within- vs. between-domain shifts and reveal that these patterns occur early, are highly dynamic, are broadly distributed, and may impact later behavior.

### Successful Decoding of External vs. Internal States.

Having uncovered evidence for distinct patterns of brain activity during shifts of attention within vs. between domains, we investigated whether and when the brain differentiated the attended domain before and after the shifts occurred. As we noted previously, owing to our experimental design, decoding of the currently attended domain (external vs. internal) was orthogonal to the above decoding of within- vs. between-domain shifts.

**Fig. 3.**
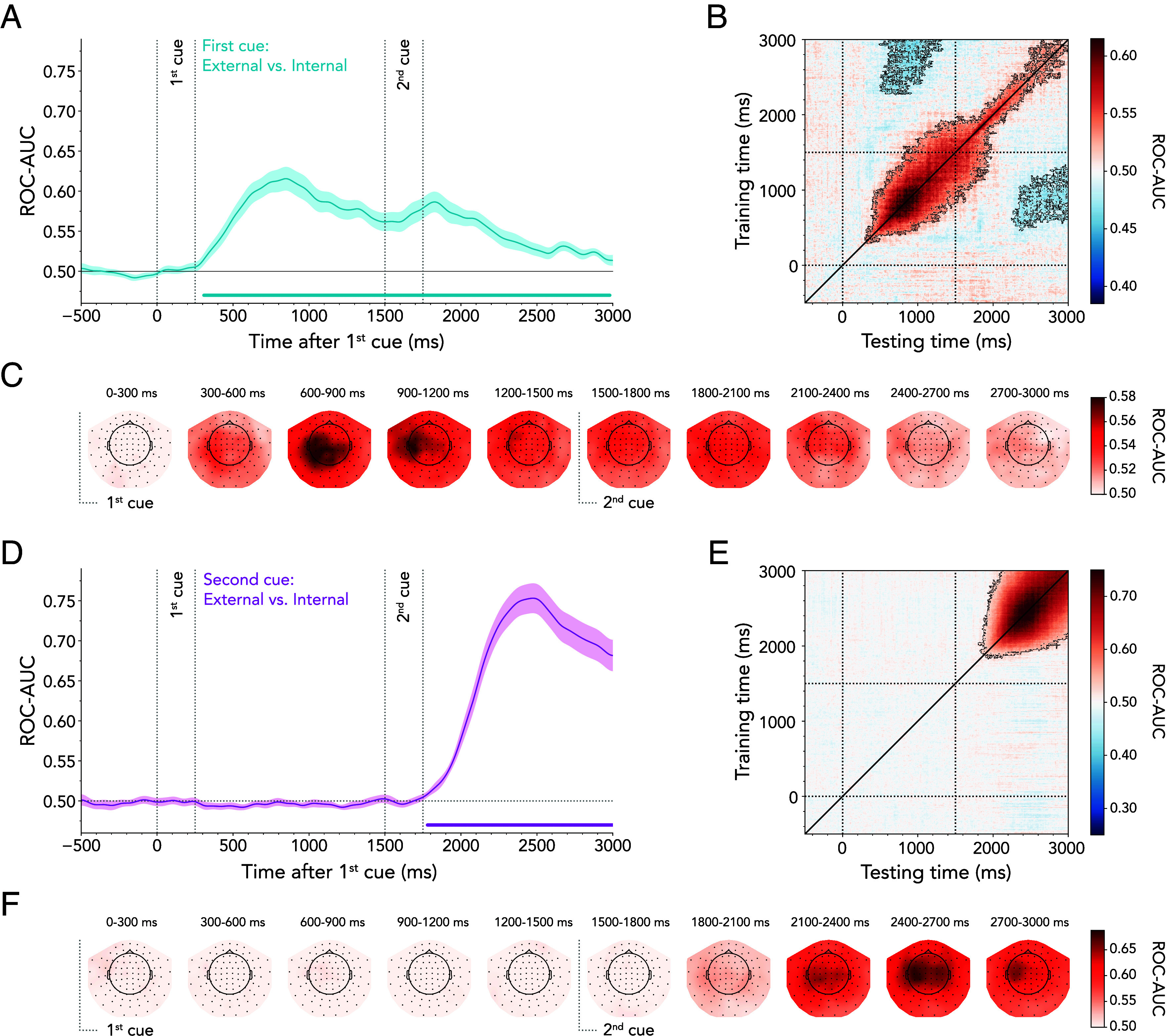
Time-resolved MEG decoding of external vs. internal attention. (*A*) Average classifier performance for distinguishing external from internal first cues plotted over time. Time course shows M ± SEM across participants. Cluster-permutation corrected significant time points are indicated with horizontal lines. (*B*) Temporal-generalization matrix reflecting classifier performance in discriminating the first-cued attentional domain. The diagonal corresponds to (*A*). Black outlines indicate significant decoding clusters. (*C*) The MEG topography averages in 300-ms steps showing which sensors most strongly contribute to the overall decoding performance in discriminating external- from internal first cues. (*D*) Same as (*A*) but based on second-cue labels. (*E*) Same as (*B*) but based on second-cue labels. (*F*) Same as (*C*) but based on second-cue labels.

We started our investigation by decoding external vs. internal attentional states, based on the labels of the first cue. To this end, we trained a classifier to distinguish external first-cue trials (i.e., external-to-external, external-to-internal) from internal first-cue trials (i.e., internal-to-internal, internal-to-external). Fig. 3*A* shows successful decoding of the attentional domain from approximately 310 ms after first-cue onset (cluster *P* < 0.001). Interestingly, the significant decoding period extended into the second-cue phase, revealing that information about the initially cued attentional state persisted throughout the delay following the second cue.

Temporal-generalization analysis unveiled an off-diagonal shape of generalization following the first cue, indicating temporal stability of the neural pattern evoked by the initially cued domain (Fig. 3*B*; cluster *P* < 0.001). In contrast, after the second cue, decoding performance occurred mainly along the diagonal, with a narrower spread to neighboring timepoints. In addition, we found off-diagonal below-chance decoding (i.e., the classifier systematically guessed incorrectly; 1st cluster *P* = 0.022, 2nd cluster *P* = 0.023). The searchlight decoding analysis demonstrated that sensor contributions to the external vs. internal decoding were broadly distributed, with peaks observed in the posterior and left-central sensors between 600 and 1,200 ms (Fig. 3*C*).

To determine whether the decoding of the first-cued attentional domain was predominantly influenced by a particular trial type, we employed two additional classifiers: one to distinguish between external-to-external and internal-to-external shifts and another to distinguish between internal-to-internal and external-to-internal shifts. *SI Appendix*, Fig. S4*A*, shows that classifier performance did not differ between these two decoding analyses, thus resembling the general external- vs. internal-domain decoding time course shown in Fig. 3*A*. In addition, we again took precautions to ensure that eye movements did not drive the external vs. internal first-cue MEG decoding results. The strength of the decoding based on gaze position was relatively weak and far from sufficient to explain the effects when decoding brain activity (*SI Appendix*, Fig. S2*E*). Furthermore, exclusion of participants who exhibited the highest mutual information between eye position and MEG decoding did not alter the MEG decoding results (*SI Appendix*, Fig. S2 *F*–*H*).

Next, we aimed to decode the domain indicated by the second cue. To this end, we trained a classifier to distinguish between external second-cue trials (i.e., external-to-external, internal-to-external) vs. internal second-cue trials (i.e., internal-to-internal, external-to-internal). Our results revealed significant decoding of the second-cued attentional domain from approximately 280 ms after second-cue onset (i.e., 1,780 ms after first-cue onset; cluster *P* < 0.001; Fig. 3*D*). As intended by our design, the second-cued domain could not be decoded before the onset of the second cue. The temporal-generalization matrix in Fig. 3*E* showed a temporal spread, implying some stability in the activation pattern (cluster *P* < 0.001). Similar to the first-cue analysis, the searchlight analysis indicated a distributed pattern of sensor contributions to decoding external vs. internal attentional domains (Fig. 3*F*).

To ensure that the results of the domain decoding were not driven by the contrast between specific trial types, we trained one classifier to discriminate between external-to-external and external-to-internal shifts and another one to discriminate between internal-to-internal and internal-to-external shifts. While both decoding time courses reached significance following the second cue, the internal-to-internal vs. internal-to-external contrast reached significance earlier than the external-to-external and external-to-internal contrast (*SI Appendix*, Fig. S4*B*). Hence, these findings revealed earlier domain differentiation when initially cued about the internal domain compared to when previously cued about an external item. In line with the first-cue analysis, we ensured that the second-cue MEG decoding results were not dependent on eye movements (*SI Appendix*, Fig. S2 *I*–*L*).

### Lingering First-Cued Attentional Domain Is Linked to Within- vs. Between-Domain Shifting.

Our results demonstrate that attentional domains indicated by the first and second cue could be decoded. Interestingly, the significant decoding of the first-cued attentional state persisted even after the second domain was cued. Supplementary analyses revealed that during the period following the second cue, the decodability of the continued information concerning the first cue was not contingent on the quality of processing of the second cue (*SI Appendix*, Fig. S5). Hence, this finding suggests that lingering processing related to the attentional domain of the first cue and instantiating the attentional domain associated with the second cue proceeded in tandem. In other words, information regarding the past and current attentional state coexisted after the second cue.

**Fig. 4.**
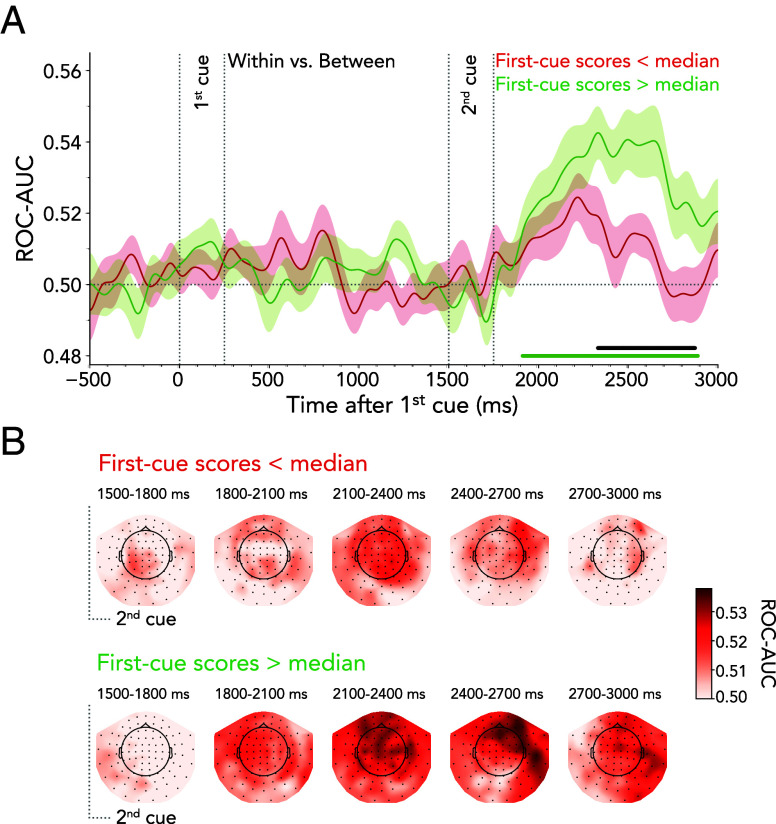
Relationship between lingering first-cued attentional domain and shift type. (*A*) Time-resolved average classifier performance for discriminating within- from between-domain shifts as a function of bad (red) vs. good (green) decoding of the lingering first-cued attention state. Time courses show M ± SEM across participants. Horizontal lines indicate significant clusters, the black line denotes difference between decoding time courses. (*B*) MEG topography averages in 300-ms steps showing which sensors most strongly contribute to the overall decoding performance in discriminating within- from between-domain shifts. *Top* panel: Trials with low decodability of the lingering first-cued domain. *Bottom* panel: Trials with high decodability of the lingering first-cued domain.

Previous task-switching research has argued that switch costs can arise from interference from prior, but now task-irrelevant, information (36, 37). We, therefore, tested whether a similar interaction might arise between lingering activity related to the first-cued domain and the ability to decode within- vs. between-domain shifts. To this end, we median-split our data into trials with good vs. bad first-cued domain decoding following the onset of the second cue (i.e., 1,500 to 3,000 ms). Next, we trained a classifier to distinguish within vs. between-domain shifts in the two resulting data subsets. The within- vs. between-domain shift type decoding only yielded a significant cluster in trials with high decodability of the lingering first-cued domain (Fig. 4*A*; ~1,910 to 2,890 ms, cluster *P* < 0.001). This was corroborated by a direct comparison, showing that trials with stronger lingering first-cue decodability had significantly more successful within- vs. between-domain decoding (~2,330 to 2,870 ms, cluster *P* = 0.005). Fig. 4*B* displays the within- vs. between-shift decoding topographies for low (*Top* panel) and high (*Bottom* panel) lingering first-cue decoding.

In addition, we found tentative evidence suggesting that the decoding quality of the lingering first-cued domain influenced the behavioral between-domain shift cost (*SI Appendix*, Fig. S6). Our data revealed a significant shift cost only when comparing trials with high classifier performance [*t*_(24)_ = 2.494, *P* = 0.020, *d* = 0.499], while no difference emerged between shift types when considering trials with low classifier performance [*t*_(24)_ = 0.945, *P* = 0.354, *d* = 0.189]. However, a direct comparison of the shift cost between low and high lingering-domain decoding did not yield significant results [*t*_(24)_ = 1.287, *P* = 0.210, *d* = 0.257].

Overall, our results suggest that the extent to which the first-cued state lingers is associated with the classifier’s ability to differentiate within- from between-domain shifts and may also predict subsequent behavioral shift costs.

### Spatial Shifts of External and Internal Attention as Tracked by Alpha Lateralization.

Thus far, we have investigated attentional shifting from two essential perspectives. First, we contrasted brain activity when transitioning between domains vs. within a single domain. Second, we examined whether brain activity distinguished between attentional domains following the first and second cues. Our task design additionally enabled us to investigate attentional shifts from a third perspective, involving the spatial orienting of attention between locations. We intentionally designed our task to guarantee that shifts occurred between items positioned in distinct hemifields, enabling us to investigate the lateralization of MEG activity according to the location to which attention was shifted.

A canonical marker of spatial attention shifts is the lateralization of posterior 8 to 12 Hz alpha activity. Contra- vs. ipsilateral attenuation of 8 to 12 Hz alpha oscillations has been linked to relative increases in excitability in sensory brain areas resulting from shifts of spatial attention in anticipation of relevant sensory stimuli as well as during selection of relevant working-memory content (14, 30, 38–42). Therefore, we focused on alpha lateralization to delineate spatial shifts of attention after external and internal first and second cues. These additional analyses complement our primary findings from the decoding analyses and help relate our work to the existing neuroscience literature on external and internal spatial shifts of attention.

**Fig. 5.**
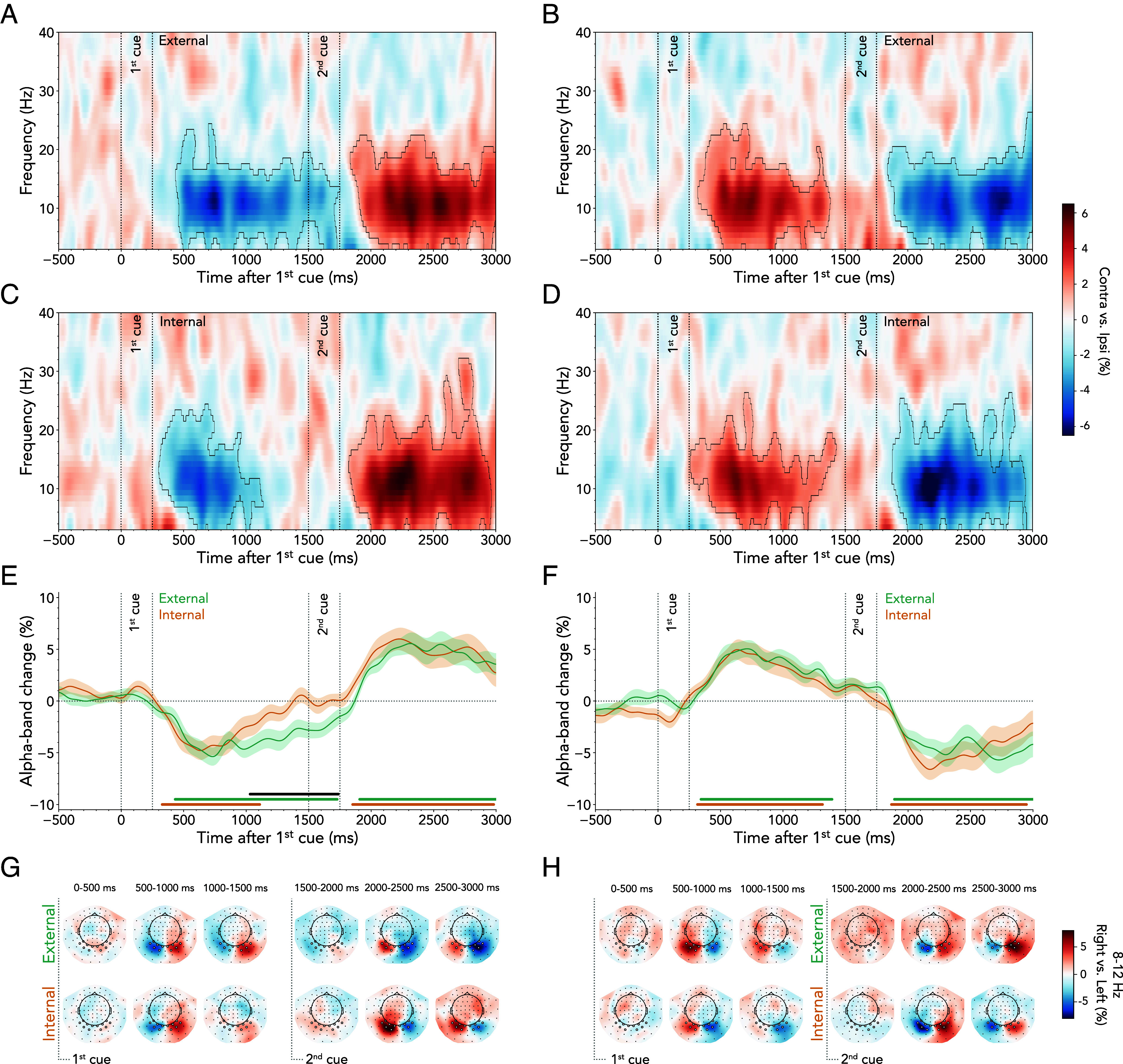
Lateralized neural activity following external and internal cues. (*A*) Time-frequency plots of neural lateralization following external first cues. Plots show the difference in spectral power contra- vs. ipsilateral to the cued side, extracted over the predefined left and right sensor clusters. Black outlines indicate significant clusters. (*B*) Same as (*A*) but for external second cues. (*C*) Same as (*A*) but for internal first cues. (*D*) Same as (*A*) but for internal second cues. (*E*) Time courses of neural lateralization in the 8 to 12 Hz alpha band relative to the first cue. Time courses show M ± SEM across participants. Horizontal lines indicate significant clusters, black line denotes difference between time courses. (*F*) Same as (*E*) but relative to second cues. There was no significant difference between time courses. (*G*) Topographies of power differences following right- vs. left-directing external first cues in the 8 to 12 Hz alpha band. The *Top* panel shows topographies for external first cues, *Bottom* panel for internal first cues. (*H*) Same as (*G*) but for second cues.

The Fig. 5 *A* and *C* shows the time- and frequency-resolved lateralization of spectral power in contra- vs. ipsilateral visual sensors for external and internal first cues, respectively. At the time of the second cue, participants were always required to shift attention from one hemifield to the other. Consequently, relative suppression of contralateral activity in response to the first cue was followed by relative enhancement of contralateral activity (i.e., a reversal of lateralization) after second-cue onset (external first cued: 1st cluster *P* < 0.001, 2nd cluster *P* < 0.001; internal first cued: 1st cluster *P* < 0.001, 2nd cluster *P* = 0.004).

To chart the time course of alpha lateralization, we averaged the time-frequency data along the classical alpha band (8 to 12 Hz; Fig. 5*E*). We confirmed the typical contra- vs. ipsilateral alpha attenuation following both external and internal first cues. Clusters of lateralized alpha attenuation alternated between hemispheres after the first (external first cued: ~430 to 1,730 ms, cluster *P* < 0.001; internal first cued: ~330 to 1,110 ms, cluster *P* < 0.001) and second cue (external first cued: ~1,910 to 3,000 ms, cluster *P* < 0.001; internal first cued: ~1,850 to 2,980 ms, cluster *P* = 0.004). The time course of the initial contra- vs. ipsilateral alpha attenuation was similar for both external- and internal first-cue trials. However, in line with prior research, the duration of the alpha modulation differed (14, 43). Lateralized alpha suppression was more transient following internally directing first cues and more sustained following externally directing first cues, as indicated by a significant cluster from approximately 1,030 to 1,740 ms after first-cue onset (cluster *P* < 0.001). A topographic inspection of external and internal first-cue trials confirmed a clear posterior topography for these effects (Fig. 5 *G*, *Upper* and *Lower* panel, respectively).

The same time-frequency analysis was conducted based on the attentional domain associated with the second cue. Fig. 5 *B* and *D* illustrates that the relative suppression of contralateral activity in response to the second cue was preceded by a relative enhancement of contralateral activity (i.e., a reversed lateralization) after first-cue onset (external second cued: 1st cluster *P* < 0.001, 2nd cluster *P* < 0.001; internal second cued: 1st cluster *P* = 0.003, 2nd cluster *P* < 0.001). As seen in the alpha-band time-courses (Fig. 5*F*), there was no difference between alpha-band lateralization following the first cue (external second cued: ~340 to 1,390 ms, cluster *P* < 0.001; internal second cued: ~320 to 1,320 ms, cluster *P* = 0.002) or following the second cue (external second cued: ~1,890 to 3,000 ms, cluster *P* < 0.001; internal second cued: ~1,870 to 2,950 ms, cluster *P* < 0.001). Topographies showing the difference between trials with right- vs. left-cued items confirmed that these visual signatures were prominent in the corresponding right and left visual sensors (Fig. 5 *H*, *Top* panel: external cue, *Bottom* panel: internal cue).

Together, these complementary analyses of alpha lateralization reveal that attention lingers longer after the first cue when directed externally vs. internally, while reorienting after the second cue proceeds similarly regardless of whether spatially reorienting to an internal or external item.

### Alpha Lateralization Proceeds Similarly During Within- and Between-Domain Shifts.

Given the effectiveness of lateralized alpha attenuation in tracking the spatial orienting and reorienting of attention, we finally turn to the important question of whether spatial orienting following the second cue would be delayed when shifting attention between as compared to within domains. If shifting between domains engages additional processes prior to instigating a spatial shift, we would expect the onset of spatial reorienting to be delayed when shifting attention between domains.

**Fig. 6.**
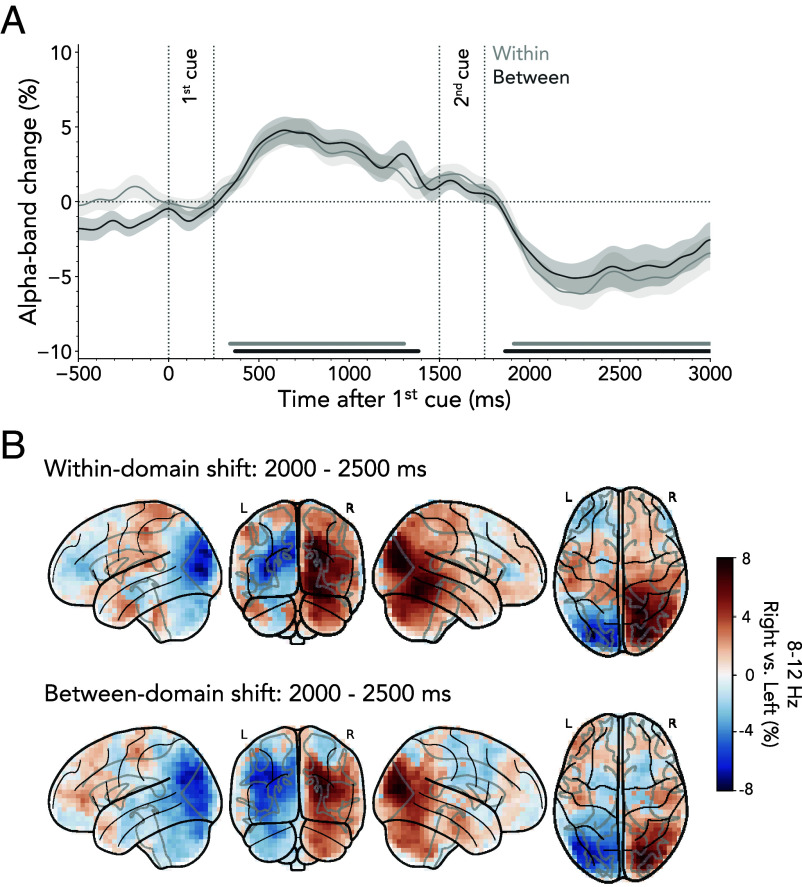
Lateralized neural activity during within- and between-domain shifts. (*A*) Time courses of neural lateralization in the 8 to 12 Hz alpha band relative to second cues indicating within- vs. between-domain shifts. Time courses show M ± SEM across participants. Horizontal lines indicate significant clusters. (*B*) Participant-averaged voxelwise difference in alpha-band activity between trials in which cues indicated visual content in the right vs. left hemifield. The *Top* panel shows source-reconstructed alpha-band activity during within-domain shifts, while the *Bottom* panel illustrates activity during between-domain shifts.

The Fig. 6*A* depicts the time course of alpha lateralization relative to the second cue, split by whether the second cue indicated a within- or a between-domain shift (within-domain shift: 1st cluster ~340 to 1,300 ms, cluster *P* = 0.002; 2nd cluster ~1,910 to 3,000 ms, cluster *P* < 0.001; between-domain shift: 1st cluster ~370 to 1,380 ms, cluster *P* < 0.001; 2nd cluster ~1,860 to 3,000 ms, cluster *P* < 0.001). Comparing time courses revealed no differences in lateralized alpha-band activity between shift types. Moreover, for both within- and between-domain shifts, source localization confirmed that the difference between trials with right- vs. left-cued items originated in right and left posterior cortices (Fig. 6 *B*, *Upper* panel: within-domain shift, *Lower* panel: between-domain shift). These findings suggest that the initiation of the spatial shift remained unaffected, despite our decoding analyses revealing distinguishable neural processing engaged during within vs. between-domain shifts.

This conclusion was further reinforced by the analysis of lateralized ERFs from posterior sensors, which revealed N2pc-like effects after both within- and between-domain shifts. These lateralized N2pc-like effects were equivalent for both shift types (*SI Appendix*, Fig. S7).

Taken together, our data provide no evidence for an additional stage invoked by between-domain shifts that must be resolved before attention can shift in space.

## Discussion

Our study uncovers direct neurophysiological evidence for distinct brain activity when shifting between perception and working-memory contents compared to when shifting between contents within either domain. The carefully balanced task design—which orthogonally manipulated shift type and target domain while also necessitating spatial shifts of attention—offered valuable insights into the dynamic neural bases that govern shifts between perception and working memory.

In principle, multiple scenarios can explain the behavioral costs and neural differences between shift types, and these need not be mutually exclusive. One obvious scenario is the addition of a supraordinate supervisory control process that acts upon the neural systems that control between-domain attentional shifts (22, 23, 44, 45). This supraordinate control mechanism could be invoked when initiating a shift between perception and working memory (and vice versa) but not when shifting between contents within either domain. Although it is most intuitive to consider a supraordinate control mechanism occurring when attention shifts between domains are initiated, it is also possible to imagine supraordinate control mechanisms operating at other stages of attentional deployment. Attention comprises several functions to guide adaptive behavior, such as disengaging, orienting, selecting, prioritizing, and output gating (46, 47). Accordingly, additional supraordinate mechanisms could operate at any or multiple points along the way. An alternative type of scenario invokes no additional control process(es). Instead, the systems for controlling external vs. internal attention may coexist and compete for access to the relevant sensory and/or motor representations. In this scenario, the additional cost of shifting between perception and memory (and vice versa) would arise from resolving a stronger degree of competition between as compared to within domains. As we outline below, our findings offer important initial clues regarding the factors that distinguish within- and between-domain shifts.

We unequivocally demonstrated distinct neural processes during within- and between-domain shifts. By tracking neural dynamics, we showed that differences occur early following the second cue (Fig. 2), suggesting they are unlikely to emerge exclusively at the response stage. In line with this, our data provided tentative evidence suggesting that the degree to which shift types can be decoded may have behavioral relevance, impacting later between-domain shift costs in RTs (*SI Appendix*, Fig. S3). We cautiously argue, therefore, that previously observed between-domain shift costs in performance (16–20, 23, 48) are unlikely explained exclusively by interference at the late response-level stage, but rather emerge already during the attention-shifting process itself.

Interestingly, by analyzing alpha-band lateralization, we found no evidence indicating differences in visual activity when orienting spatial attention within vs. between domains (Fig. 6 and *SI Appendix*, Fig. S7 for lateralized ERFs). This observation challenges the intuitive scenario proposing that a supraordinate control mechanism is invoked when initiating an attentional shift that crosses domains. Such a scenario would predict a delayed shift of spatial attention. We found no evidence for this scenario. Spatial orienting and selection functions of attention proceeded with equivalent time courses, as reflected by lateralized modulations of posterior alpha and ERFs. These findings complement a similar observation that between-domain shifts did not alter patterns of gaze biases associated with spatial shifts (17), further suggesting that no additional, time-consuming gating step occurs before spatial shifts between domains.

Neural patterns differentiating within- vs. between-domain shifts evolved relatively dynamically and were broadly distributed (Fig. 2 *B* and *C*). Differences between shift types are thus not solely attributable to a single tonic state that differs between shift types but likely result from the dynamic interplay of multiple states. Moreover, the distributed nature of the within- vs. between-shift decoding suggests that large-scale networks may be involved. It will be important for future brain-imaging investigations using functional MRI (fMRI) and/or animal models to substantiate this broad distribution and to pinpoint the precise spatial underpinnings of the here-reported findings.

It is useful to consider that each within- and between-domain shift is a consequence of the attended domains before and after the shift. Therefore, to understand the nature of the two shift types, it is also essential to investigate the neural dynamics evoked by the first- and second-cued attentional domains. While some studies have emphasized the shared neural systems and dynamics between selection mechanisms in external and internal attention (10, 15), others have highlighted important differences (7, 11, 46, 47, 49–53). Underscoring the differences, our data demonstrate successful decoding of external vs. internal attention (Fig. 3). Moreover, we show that decoding of the first-cued domain persisted even after the presentation of the second cue.

The carry-over decoding effect of the first-cued domain occurred regardless of the domain indicated by the second cue (*SI Appendix*, Fig. S4*A*). This finding highlights that the attentional state invoked by the second cue did not overrule or wipe out the lingering state invoked by the first cue. Multiple processes could have contributed to the lingering decoding of the first-cued domain, and we are unable to adjudicate among these without further investigation. For example, anticipating the spatial location or sensory features of a target in the external domain may have elicited sustained neural activity according to spatial or feature-based receptive field properties (54–56). In contrast, cueing internal representations in working memory often triggers more transient sensory modulation (14, 43). Our alpha-lateralization findings hint at these effects, with more prolonged modulation following spatial cues indicating targets in the external than internal domains (Fig. 5 *A*, *C*, *E*, and *G*). Alternatively, the presentation of the second cue may have activated latent functional states in working memory through a pinging mechanism (57–59). This neural reactivation can also occur in the presence of active working-memory representations, causing an increase in their decodability (60). Although the second cue indicated a different item than the first cue, its associated sensory input may have interacted with latent or activated working-memory states, potentially acting as a ping in trials in which attention was initially directed to an internal item. Another possibility is that the first cue may have placed the system in different states of sensorimotor preparation. If an internal item was cued, its representation could have been readily transformed into a motor code (29, 30, 61), whereas this was not possible when sensory items were anticipated by external attention. Therefore, the sustained decoding of the first-cued attentional domain following the second cue could reflect a lasting effect of output gating following internal but not external first cues. Any, or all, of these differences may have contributed to the successful decoding of the first-cued domain after the second cue.

The decodability of the lingering first-cued attentional domain did not trade off against the decoding of the second-cued attentional domain (*SI Appendix*, Fig. S5). Instead, after the second cue, information processing unfolded for both the first- and second-cued attentional domains in tandem, implying the coexistence of preceding and current attention states in the brain. Though our data do not directly tap into the nature or potential functional relevance of these coexisting states, they did reveal that the degree to which the first state lingered (i.e., could still be decoded after the second cue) predicted how well our classifier could distinguish within- from between-domain shifts (Fig. 4). Moreover, our data offered tentative evidence that lingering activity from the first cue predicted the behavioral between-domain shift cost (*SI Appendix*, Fig. S6). This suggests that preceding attentional states influence upcoming attentional shifts. What role such lingering states play, and how parallel processing of contents from preceding and current attention states is mediated, remains an exciting endeavor for future studies.

Our findings and task design pave the way for investigating the dynamic mechanisms orchestrating the seamless shifts of attention between external and internal contents to guide adaptive flexible behavior. We offer an initial exploration, opening the door to relevant unanswered questions and encouraging further task manipulations. Many additional interesting factors and dimensions remain to be considered. Additional manipulations will yield complementary insights and contribute to a more comprehensive understanding of the principles and mechanisms that govern attention shifts between perception and working memory in service of adaptive behavior.

One important factor to consider is that external and internal attention can be operationalized in different ways. In our study, we exploited pre-cues and retro-cues as tools to orient attention toward anticipated near-future external stimuli or near-past sensory stimuli maintained in working memory, respectively. In this conceptualization and task design, participants can select relevant contents from working memory when cued internally but must await the incoming stimulation before selecting contents when cued externally. However, external attention is not limited to anticipating sensory events but also operates when sensory content is available for immediate selection, such as during visual search. In future studies, it will be interesting to investigate the dynamics of transitioning between external and internal content in tasks where external information is presented concurrently with external cues and remains perceptually available until the reporting stage.

Moreover, the informative cues used in this study engaged goal-directed processes. The source of attentional control was thus inherently endogenous (i.e., internal) for both externally and internally directed cues. It is important to note that the core distinction between external and internal attention concerned the stimulus contents to be selected and prioritized: upcoming sensory stimulation or maintained memory representations. Future research can also compare how various sources of attention guide between-domain shifts.

Finally, in our study, all double-cue trials required a spatial shift of attention when the second cue was presented. This design characteristic ensured that external and internal attention conditions were optimally balanced and minimized potential interference from items occurring at the same location. Future studies can investigate whether and how spatial overlap and proximity interact with shifting attention between domains.

In summary, we revealed distinct neural dynamics for shifting within vs. between perception and working-memory contents. We demonstrated how these distinct neural processes emerge quickly, evolve dynamically, are broadly distributed, and occur without delaying the timing of spatial orienting of attention. Besides successfully decoding the shift type, we were also able to decode the attentional domain both before and after the shift, thereby capturing the origin and destination of attention. Our results uncovered a direct link between the previously attended domain and the performed shift type, suggesting that lingering activity related to the first-cued domain affects processing following the second cue in a way that distinguishes within- from between-domain shifts. Brain imaging methods with higher spatial resolution should build on these findings to identify the brain networks that orchestrate shifts of attention between domains and investigate how they interact with the more established control networks involved in modulating shifts of attention within the external and internal domains.

## Materials and Methods

A detailed overview of the methods employed in this study is provided in *SI Appendix*, *Methods and Materials*. This section describes our sample of 25 participants, outlines the experimental design, and details the methods used to acquire behavioral measures, eye-tracking data, MEG recordings, and structural MRI scans. Additionally, this section offers comprehensive information on the analyses performed. In our behavioral analysis, we compared the effects of target domain (external vs. internal) and shift type (within vs. between) on RTs and reproduction errors. For the MEG analysis, we first preprocessed and epoched the sensor-level MEG data before applying various analysis approaches. We employed time-resolved multivariate pattern analyses and ERF analyses to investigate neural activity related to external vs. internal attention and within- vs. between-domain shifts. To explore the relationship between decoding results and behavioral outcomes, we employed a median-split approach on trial-wise decoding scores and subsequently included the median-split factor in our behavioral analysis when comparing conditions. Moreover, we conducted decoding analyses on the eye-tracking data and computed mutual information to assess the impact of eye movements on the MEG decoding results. Finally, we applied time-frequency analyses, ERF analyses, and source localization to examine differences in spatial attention shifts across target domains and shift types. Statistical analyses of the behavioral data were conducted using repeated measures ANOVA and *t* tests, while cluster-based permutation tests were applied to the MEG data.

## Supplementary Material

Appendix 01 (PDF)

## Data Availability

Data, Materials, and Software data have been deposited in OSF (https://doi.org/10.17605/OSF.IO/5CW7U) (62).
