# Supplementary material for: Neural dynamics of shifting attention between perception and working-memory contents
Source: Proc Natl Acad Sci U S A. 2024 Nov 13;121(47):e2406061121. doi: 10.1073/pnas.2406061121 (PMC11588118; doi:10.1073/pnas.2406061121)
Supplement: Supplementary file 1 — Appendix 01 (PDF) [file pnas.2406061121.sapp.pdf]

PNAS

Supporting Information for

Neural dynamics of shifting attention between perception and working-memory contents

Daniela Gresch[a,b,c,1], Sage E.P. Boettcher[a,b], Chetan Gohil[b], Freek van Ede[d], Anna C. Nobre[a,b,c,e,1]

[a] Department of Experimental Psychology, University of Oxford, Oxford, OX2 6GG, United Kingdom

[b] Oxford Centre for Human Brain Activity, Wellcome Centre for Integrative Neuroimaging, Department of Psychiatry, University of Oxford, Oxford, OX3 7JX, United Kingdom

[c] Department of Psychology, Yale University, New Haven, CT 06510, United States of America

[d] Institute for Brain and Behaviour Amsterdam, Department of Experimental and Applied Psychology, Vrije Universiteit Amsterdam, 1081 HV, Amsterdam, The Netherlands

[e] Wu Tsai Institute, Yale University, New Haven, CT 06510, United States of America

[1] To whom correspondence may be addressed. Email: daniela.gresch@yale.edu or kia.nobre@yale.edu

This PDF file includes

- Methods and Materials
- Figures S1 to S7
- Table S1
- SI References

Methods and Materials

Participants

The study was approved by the Central University Research Ethics Committee of the University of Oxford. The sample size was set to *n* = 25, based on a previous study from our lab targeting a similar research question via behavioral measures (1). To yield the targeted number of participants, we collected data from 26 participants. One participant was excluded following our a-priori behavioral trial-removal procedure (see Behavioral Analysis). Of the remaining 25 participants (age range: 19 to 36; mean age: 26.24; 14 female, 11 male), 22 reported being right-handed (three left-handed). Individuals provided written informed consent before participating in the study and were paid £15 per hour.

Task and Procedure

The current task design builds on our previous behavioral study (1). In each trial, four randomly oriented bars appeared within colored circular placeholders at quadrant locations. Early in the trial, two oriented bars were bilaterally presented and had to be encoded into working memory. Later in the trial, two bars appeared briefly in the remaining placeholders followed by masks. Between the initial and final presentation of the bars, one or two centrally presented color cues signaled which of the four bars participants had to report by accurately reproducing its exact orientation. Cues that indicated a previously encoded bar directed attention internally (retro-cues or internal cues), indicating which item from working memory should be reported. In contrast, cues that indicated a previously unoccupied placeholder directed attention externally (pre-cues or external cues), specifying which item from the upcoming perceptual array should be reported.

At the start of each trial, a white central fixation cross (RGB value: [255, 255, 255]) surrounded by four colored circular placeholders appeared against a black background (RGB value: [0, 0, 0]) for 750 ms. Each of the placeholders appeared in one of four highly distinguishable colors (RGB values: light blue [0, 159, 183], orange [217, 120, 0], green [47, 167, 0], pink [247, 35, 255]). The location mapping of the four placeholder colors was randomly determined on each trial. Placeholders subtended 3.5° visual angle and were centered in the top-left, top-right, bottom-left, and bottom-right at a distance of 4.5° visual angle from fixation. Placeholders stayed on the screen until the response was required at the end of the trial.

Next, two tilted grey bars (RGB value: [50, 50, 50]) subtending approximately 0.5°×3.4° appeared for 500 ms inside two of these placeholders (referred to as internal display). The orientation of the bars was randomly determined between 0 and 180°. Participants were instructed to memorize the orientation of these two bars. There were four possible configurations of the internal display, which occurred equally often throughout the experiment: the bars could appear in the placeholders located in the (1) top-left and top-right, (2) bottom-left and bottom-right, (3) top-left and bottom-right, and (4) top-right and bottom-left. As such, at the time of the internal display, there was always one bar presented in each visual hemifield.

The internal display was followed by a delay of 750 ms, during which only the fixation cross and placeholders remained on the screen. The central fixation cross then changed color to match that of one of the placeholders for 250 ms. The color change acted as an informative cue, indicating the potential item to be reported. This cue could indicate a placeholder that previously contained an item during the internal display (i.e., retro-cue) or a placeholder in which no item had been presented yet (i.e., an item of the upcoming external display; pre-cue). The cued location and attentional domain were pseudo-randomized such that each of the four placeholder locations and each domain was equally often indicated by the cue.

In 50% of the trials, the central fixation cross changed color again after 1250 ms to match the color of another placeholder for 250 ms (i.e., double-cue or shift trials). In the remaining 50% of the trials, only a single cue (i.e., single-cue or stay trials) was presented. In single-cue trials, the cue appeared at the same time as the first cue in double-cue trials and was 100% instructive, always indicating the target for report. Single-cue trials were included to increase the relevance of the first cue

and ensure that participants shifted attention to the first-cued item rather than simply ignoring the first cue entirely. Single-cue trials served no other purpose for the behavioral and neural comparisons relevant to the research question addressed in the present study.

The double-cue trials were the important trials for our research question since these trials required a shift of attention. In particular, the double-cue condition was further divided into two sub-conditions: within- and between-domain shifts. In within-domain shift trials (i.e., external-to-external or internal-to-internal), the second cue indicated the alternative item within the same domain as the first cue occurring in the other hemifield. In between-domain shift trials (i.e., external-to-internal or internal-to-external), the second cue indicated the item in the alternative domain occurring in the other hemifield. Thus, in within- and between-domain shift trials, attention always needed to be reoriented from one hemifield to the other. The second-cued location and domain were pseudo-randomized such that each of the four placeholder locations and each domain were equally often indicated. In double-cue trials, the second cue instructed the target item for report with a certainty of 100%, thus overruling the first cue. Accordingly, the target item was always identical to the second-cued item. The color of the first and second cue always differed in double-cue trials.

The single cue in single-cue trials and the second cue in double-cue trials were followed by an inter-stimulus interval of 1250 ms before the onset of a perceptual display (referred to as external display), containing two randomly tilted bars. The new items appeared in the yet-unoccupied placeholders for 50 ms. For example, if the bars in the internal display appeared in the top-left and bottom-right placeholders, bars in the external display occurred the top-right and bottom-left placeholders. To make the perceptual discrimination challenging, items in the external array were masked. The masking array consisted of four overlayed titled bars presented in each of the placeholders for 100 ms. At each mask location the four tilted bars differed 45° from the neighboring bar. The overall orientation of each mask was randomly drawn at 0 ms and 50 ms, thus creating the impression of a dynamic display. The masking parameters were set to equate the difficulty of external and internal reports, based on piloting efforts.

The offset of the dynamic mask was followed by a 100-ms delay, after which a visual response dial was displayed, always starting in a random position. The response dial surrounding the fixation cross was the same size as the placeholders and included markers along a circle that corresponded to the ends of a bar. The dial rotated leftwards when pressing the left button and rightwards when pressing the right button of the response device held in the dominant hand. The dial rotated with a speed 1/10° per millisecond. Participants had unlimited time to complete the orientation report. When the dial aligned with the orientation of the target item, participants pressed either of the two buttons of the response device held in the non-dominant hand to confirm their response and continue with the task.

After a 100-ms delay in which only the fixation cross stayed on the screen, participants received feedback in the form of a number ranging from 0 to 100, with 100 indicating a perfect report and 0 indicating that the adjusted orientation was perpendicular to the angle of the target item. Feedback was presented 0.7° above the central fixation cross for 300 ms. Trials were separated by an inter-trial interval randomly drawn between 750 and 1000 ms. Between blocks, participants were presented with their average reproduction accuracy in the previous block.

The experiment consisted of 512 trials divided across 8 blocks, each including 64 trials. Of the total trials, 50% (256) were single-cue trials and 50% (256) were double-cue trials. Participants were equally likely to report an external or internal item in single-cue trials (128 each). Double-cue trials varied in the type of shift performed, that is, 50% (128) required a within-domain shift and the other 50% (128) a between-domain shift. Within-domain and between-domain shift trials were further split by the target domain. Ergo, the within-domain shift trials included 50% (64) external-to-external and 50% (64) internal-to-internal shifts, while the between-domain shift trials included 50% (64) external-to-internal and 50% (64) internal-to-external shifts. In each of the four unique shift conditions, participants were equally likely to shift their spatial attention between any of the eight possible cross-hemifield combinations of placeholders. The orthogonal manipulation of the to-be-reported target

domain (external vs. internal) and the shift type (within-domain shift vs. between-domain shift) enabled us to independently investigate these two factors. Each block consisted of 50% (32) single- and 50% (32) double-cue trials.

To become familiarized with the procedure of the experiment, participants performed a practice block before starting the data collection. The whole experiment lasted approximately 65 min.

Data Acquisition

We obtained whole-head MEG recordings in a magnetically shielded room using a 306-sensor MEG system (204 first-order planar gradiometers, 102 magnetometers; TRIUX neo, MEGIN OY, Espoo, Finland) at the Oxford Centre for Human Brain Activity. The MEG signal was sampled at 1000 Hz, with a high-pass filter at 0.1 Hz and a low-pass anti-aliasing filter at 330 Hz. Each experimental block was recorded separately, resulting in eight runs for most participants. However, due to technical issues, two individuals had fewer recordings, with six and seven recordings obtained, respectively.

Prior to data acquisition, we digitized the individual head shape with an electromagnetic position and orientation monitoring system (FASTRAK, Polhemus, Colchester Vermont, USA). Shapes included fiducial landmarks (nasion, right and left preauricular points) and about 250 additional points evenly spread on the participants' scalp. Five Head Position Indicator (HPI) coils were placed on participants' mastoid bones and forehead to keep track of participants' head position inside the dewar through electromagnetic induction before and after each recording block.

Participants performed the task in a seated position. Participants were instructed to avoid head, body, and limb movements and to keep strict eye fixation during the experimental blocks. In addition to electrocardiography (ECG) and horizontal and vertical electrooculography (EOG), eye positions were continuously monitored by an MEG-compatible eye-tracking device with a sampling frequency of 1000 Hz (Eyelink 1000, SR-Research Ltd., Ottawa, Ontario, Canada). At the beginning of each experimental session, participants performed an eye-tracking calibration task to verify their gaze position on the screen. Calibration was repeated if drift was noticed during the experimental session.

We used the PsychoPy package (version 2021.2.3) (2) in Python for stimulus generation and stimulus delivery. The stimuli were projected on a translucent whiteboard using a DLP LED projector (ProPixx, VPixx Technologies Inc., Saint-Bruno, Quebec, Canada) at a 120-Hz refresh rate. The whiteboard was located at 120-cm distance from the participant, and it provided a projection area of 55×31-cm and 1920×1080-pixel resolution. A bimanual fiber-optic response device was used to collect manual responses.

Structural magnetic resonance imaging (MRI) scans were acquired for 22 participants at the Oxford Centre for Human Brain Activity using a Siemens Prisma 3T scanner, either immediately following the MEG recording or on a separate day. Existing structural 3T MRI scans for three participants were obtained from previous studies following data-sharing procedures.

Behavioral Analysis

Behavioral data were analyzed in R Studio (3). During pre-processing (similar to (1)) trials were removed when RTs (calculated from dial onset to response initiation) exceeded 5000 ms. Next, we removed trials for which the remaining RTs were 2.5 *SD* above the individual mean across all conditions. Moreover, we removed datasets with average reproduction errors (calculated by averaging the absolute difference between the angle of the target item and the reported angle) equal to or higher than 45° in any of the conditions ($n = 1$). After these exclusion steps, datasets from 25 participants remained in the main behavioral analysis, with an average of 16.56 ± 3.99 (*M* ± *SD*) trials removed. Additionally, we removed the bad MEG trials from the behavioral data analysis.

To meet parametric-test assumptions, the distribution and power coefficient of all dependent variables were inspected using the MASS package (4) and the Box-Cox procedure (5). As a result, RTs and reproduction errors were square-root-transformed. For the visualization of RTs and reproduction errors, we used milliseconds and degrees as scales, respectively, while all behavioral inferential statistics were conducted on the square-root-transformed data.

The conditions of interest were target domain (having to report an external vs. internal item) and shift type (within vs. between). When comparing more than two means, we applied a repeated measures analysis of variance (ANOVA) and reported η^2_G as a measure of effect size. When evaluating only two means, we applied a paired samples *t*-test and report Cohen's *d* as a measure of effect size.

MEG Pre-processing

MEG data were analyzed in Python using MNE-Python (version 1.4.2) (6) and OSL (version 1.1.0) (7) combined with custom code. For each individual run, we applied the Elekta MaxFilter (version 2.2) implementation of Spatiotemporal Signal Source Separation to remove external noise (TSSS) (8), to continuously compensate for head movements occurring during the run, and to realign all runs per participant to a participant-specific head position. The reference head position for each participant was determined by minimizing the head position distance between runs. Next, data were downsampled to 250 Hz and low-pass filtered at 50 Hz. Importantly, no high-pass filter was applied, since high-pass filtering can temporally displace multivariate information (9). Bad sensors were identified using a generalized extreme studentized deviate (ESD) test (10) at a 0.05 significance threshold and subsequently interpolated. On average, 1.62 ± 3.11 (*M* ± *SD*) bad gradiometers and 1.42 ± 2.02 bad magnetometers were identified and interpolated per run. We applied an independent component analysis (ICA) to regress out eye-movement and cardiac-related activity. Note that the maximum number of ICA components to be extracted was determined by the residual degrees of freedom after TSSS rank reduction during Maxfiltering (11). Data were epoched from -500 to 3000 ms relative to the first-cue onset without applying baseline correction (9). Next, we combined all individual runs for each participant by concatenating the data. Additionally, we remapped magnetometers onto gradiometers, allowing us to conduct all subsequent sensor-level analyses using a unified sensor type. Lastly, we employed an ESD trial rejection approach to detect and exclude trials with high variance. Per participant, 5.24 ± 6.96 (*M* ± *SD*) trials were removed. In addition, we removed the trials that were excluded in the behavioral data analysis. All following analyses were conducted using sensor-level MEG data, unless stated otherwise.

Time-resolved Multivariate Pattern Analysis

We performed time-resolved multivariate analysis on broadband MEG data using the Scikit-learn toolbox (version 1.3.0) (12) and built-in decoding functions provided by MNE-Python (version 1.4.2) (6). The decoding pipeline closely followed the approach outlined in previous literature (13).

We extracted patterns of brain activity separately for each participant on a timepoint-by-timepoint basis (*mne.decoding.SlidingEstimator*). First, features (i.e., data at each time point for each sensor) were centered and scaled based on data from all trials (*sklearn.preprocessing.StandardScaler*). The true spatial dimensionality of the MEG data after the Maxfilter preprocessing algorithm is around 70, rather than the 204 gradiometer sensors (11). Therefore, to reduce the number of redundant features for decoding, we performed Principal Component Analysis on the standardized data while maintaining 99% of the variance (*sklearn.decomposition.PCA*). Training and testing were done on the same data using a 10-fold stratified cross-validation procedure (*sklearn.model_selection.StratifiedKFold*). First, trials were randomized and divided into 10 equal-sized folds. Next, a leave-one-out procedure was used on the 10 folds, such that the classifier was trained on 9 folds and tested on the remaining fold. This procedure was repeated 10 times until each fold was used once for testing. A LDA classifier (*sklearn.discriminant_analysis.LinearDisciminantAnalysis*) was utilized to train on the provided training data and labels. LDA is a supervised dimensionality-reduction technique that aims to find a linear combination of features that maximizes class separability, and has been recommended for neuroscientific decoding studies (14). We used the ROC-AUC as the scoring metric. This scoring metric takes into account the trade-off between true and false positive rates and is considered a sensitive, nonparametric, and criterion-free measure of classification (15, 16). A ROC-AUC value of 0.5 means chance-level classification performance. The obtained classifier scores were averaged over all 10 folds, yielding a single decoding estimate per participant, timepoint, and condition.

Lastly, decoding time courses were smoothed with a one-dimensional Gaussian filter with a *SD* of 10 samples (i.e., 40 ms).

In addition, we used temporal-generalization methods (17) to examine whether there are recurrent neural activation patterns over time, that is, whether an activation pattern observed at one timepoint is observed again at a later timepoint. Generalization off the diagonal in the temporal generalization matrix is driven by overlap in the activation pattern at two different timepoints, and therefore implies that the activation pattern recurred. Temporal generalization allows us to examine such potential overlaps in representations over time. This approach results in timepoint-by-timepoint decoding matrices in which each cell corresponds to a classification accuracy at a unique training- and test-timepoint combination.

To test which brain areas contributed most to the classifier likelihoods observed in our multivariate methods, we ran a sensor-space searchlight decoding analysis (similar to 13, 30, 34, 73), analogous to the searchlight analysis developed for functional MRI (21). For this analysis, we ran the same analysis as outlined above across small clusters of 15 neighboring MEG sensors, resulting in decoding scores for each sensor and timepoint. These decoding scores were averaged into time clusters of 300 ms each, resulting in 10 topographical maps between 0 and 3000 ms. These topographical plots show whether decoding results were primarily driven by specific sensor clusters. Note that although this approach differs from the analysis of activation patterns, it ultimately serves the same purpose: identifying the sources of neural processes in space and time (22). We did not test any specific hypotheses regarding the spatial distributions of the effects and therefore did not run any statistical tests on the results.

Even though stratified cross-validation is highly sensitive to class imbalances (16), we additionally applied between-class balancing using under-sampling prior to the decoding analysis, such that the number of trials was equal across the classes. This further ensured that during training the classifier would not only achieve a high decoding performance merely by systematically and blindly voting for the majority class (23). Note that as the design was balanced in terms of trial counts, the between-class balancing was employed to account for small imbalances due to trial-rejection procedures used for data cleaning.

Relationship Between Decoding and Behavior

To investigate the relationship between the observed decoding of within- vs. between-domain shifts of attention and the behavioral shift costs, we employed the following approach. Firstly, we identified trials with low and high classifier performance for each participant by computing timepoint-by-timepoint decoding scores and averaging them across the interval of interest. Subsequently, we performed a median split based on these average decoding scores, creating two groups of trials: one with low decoding scores and the other with high decoding scores. We then included the median-split factor in our behavioral analysis when comparing conditions.

Control Analysis: Eye Movements

Involuntary eye movements are a common problematic covariate in studies using multivariate approaches (24, 25). To test for an influence of gaze on the MEG findings, we ran the same MEG decoding analyses on our eye-tracking data. We acquired a total of 23 eye-tracking datasets, consisting of 15 binocular recordings and 8 monocular recordings. For the binocular recordings, we merged the x- and y-coordinates from both eyes to create a single metric. The data were then epoched from -500 ms to 3000 ms relative to the first-cue onset and were baseline-corrected prior to cue onset (-500 to 0 ms). Next, we trained the classifier to distinguish between different conditions at each timepoint using only the x- and y-coordinates of the eye-tracking data. Instead of a LDA classifier, we used a Support Vector Machine classifier (SVC; *sklearn.svm.SVC*) with Radial Basis Function (RBF) kernel. The reason behind opting for SVC with RBF kernel is the likelihood that the distribution of x- and y-coordinates of gaze is not linearly separable. In all conditions, participants were instructed to maintain fixation, making it challenging to distinguish between different classes or conditions using a simple linear boundary. The

RBF kernel is a powerful tool for transforming non-linearly separable data into a higher-dimensional space, making it possible to separate the data using a hyperplane (26). This method is particularly effective when dealing with datasets a smaller number of features than of samples.

By computing mutual information scores over correctly classified MEG trials on a timepoint-by-timepoint basis, we assessed how much information was shared between the MEG decoding results and eye-movement decoding results (25). Mutual information quantifies the extent to which knowledge of one variable reduces uncertainty about another variable. Higher mutual information values indicate a stronger dependence between two variables, suggesting that changes in one variable are informative about the other.

Time-frequency Analysis

We generated time-frequency representations of power by convolving the data with Morlet wavelets across the frequency range of 3 to 40 Hz. For each frequency, we used a fixed 300-ms time window such that the number of cycles changed with the frequency. The power time series in the planar gradiometer pairs were then combined (i.e., root mean square), resulting in a 102-sensor combined planar gradiometer map in sensor space.

For the comparison between contra- and ipsilateral power, we computed activity following left- and right-directing cues, separately for posterior left sensor clusters (MEG1632+1633, MEG1642+1643, MEG1912+1913, MEG1922+1923, MEG1942+1943, MEG2042+2043) and right sensor clusters (MEG2032+2033, MEG2312+2313, MEG2322+2323, MEG2342+2343, MEG2432+2433, MEG2442+2443), and subsequently pooled the contra- vs. ipsilateral contrasts between them. We expressed the contra-vs-ipsi contrast as a normalized difference [i.e., ((contra – ipsi)/(contra + ipsi)) × 100]. Sensors were chosen based on prior MEG studies from our lab that have consistently implicated the same set of posterior sensors for capturing neural activity related to lateralized visual stimuli (27, 28). While we selected these sensor clusters based on independent data, we could confirm their validity and appropriateness in the current data (**Fig. 5g** and **h** for the overlay of these sensor clusters on the relevant right vs. left topographies).

To extract time courses of alpha lateralization, we averaged the contra- vs. ipsilateral activity across the predefined alpha band (8-12 Hz) in the visual sensors. Topographies of the lateralized visual activity were obtained by contrasting trials in which cues indicated visual content in the right vs. left hemifield. To depict the topographies associated with relevant condition comparisons, we focused on the alpha frequency bands between 0 and 3000 ms in predefined steps of 500 ms.

Event-related Fields Analysis

To complement the decoding and time-frequency analyses, we performed a univariate analysis of ERFs. The sensor-level MEG data used in the ERF analysis underwent similar preprocessing as the data for the remaining analyses, with two important differences. First, to eliminate slow drifts, we applied a 1-Hz high-pass filter to the continuous data. Second, epochs were baseline-corrected using the 500-ms window preceding the onset of the first cue. As with the time-frequency analysis, planar gradiometer pairs were combined using the root mean square, resulting in a 102-sensor combined planar gradiometer map in sensor space.

The combined planar gradiometers were divided by hemisphere (left and right) and lobe (frontal, temporal, parietal, and occipital) into eight sensor clusters with 12 or 13 pairs each (**Appendix SI, Table S1**) according to (29). For each sensor cluster and shift-type condition, we computed the averaged ERF time course.

Moreover, to compare lateralized visual activity in posterior regions, we calculated separate waveforms from the left and right sensor clusters (using the same sensor clusters as in the time-frequency analysis), following both left- and right-directing cues. This resulted in four separate waveforms: two from the left sensors (i.e., cue left, cue right) and two from the right sensors (i.e., cue left, cue right]). Contra- and ipsilateral ERF time courses were then calculated by averaging the waveforms of trials wherein the cue indicated an item appearing on the opposing or same side as the

relative sensor cluster, respectively. To compare lateralized brain activity between shift types, we computed the contra- vs. ipsilateral contrast for each condition. All ERF time courses were smoothed with a one-dimensional Gaussian filter with a *SD* of 5 samples (i.e., 20 ms).

Source Localization

To visualize sources of oscillatory brain activity, sensor-level MEG scans were source reconstructed using OSL (version 1.1.0) (7). For each participant, structural MRI and MEG coordinate systems were co-registered by matching digitized anatomical fiducial landmarks and head shape points on the participant-specific T1 scans. Forward modelling used a single layer representing the inner skull surface (i.e., single-shell boundary element model). The epoched sensor-level data were reconstructed onto a 5-mm isotropic grid using a unit-noise-gain Linearly Constrained Minimum Variance (LCMV) beamformer (11, 30). The data covariance matrix used to calculate the beamformer was estimated using the continuous pre-processed sensor-level data for each participant, regularized to a rank of 60 using PCA. A diagonal matrix containing the variance of each sensor type was used as noise covariance. The beamformer provides the activity in the x-, y-, z-direction at each grid point. This was projected onto an axis that maximizes power to obtain a single time course for each grid point (i.e., voxel).

The participant-averaged voxelwise difference in alpha-band activity between trials in which cues indicated visual content in the right vs. left hemifield was plotted on the MNI152 standard brain template.

Statistical Analysis

Statistical evaluation of MEG data was conducted using a cluster-based non-parametric permutation approach with 1024 permutations and a cluster alpha of 0.05. This approach offers a solution to the challenges related to multiple comparisons in the statistical analysis of MEG data, especially when dealing with a large number of comparisons. In particular, this approach evaluates temporal clusters observed in the original (non-permuted) data against a permutation distribution derived from the largest temporal cluster found after each permutation of the condition labels (31).

In a supplementary analysis, we additionally utilized a jack-knife approach to identify differences in decoding-onset times. For the jack-knife approach, we computed decoding time-course averages by excluding each of the n data sets once, allowing us to obtain n leave-one-out grand averages. Next, we identified the time point at which the decoding time course reached 50% of its peak. Jack-knifed standard errors, t-values, and p-values were adjusted according to (32).

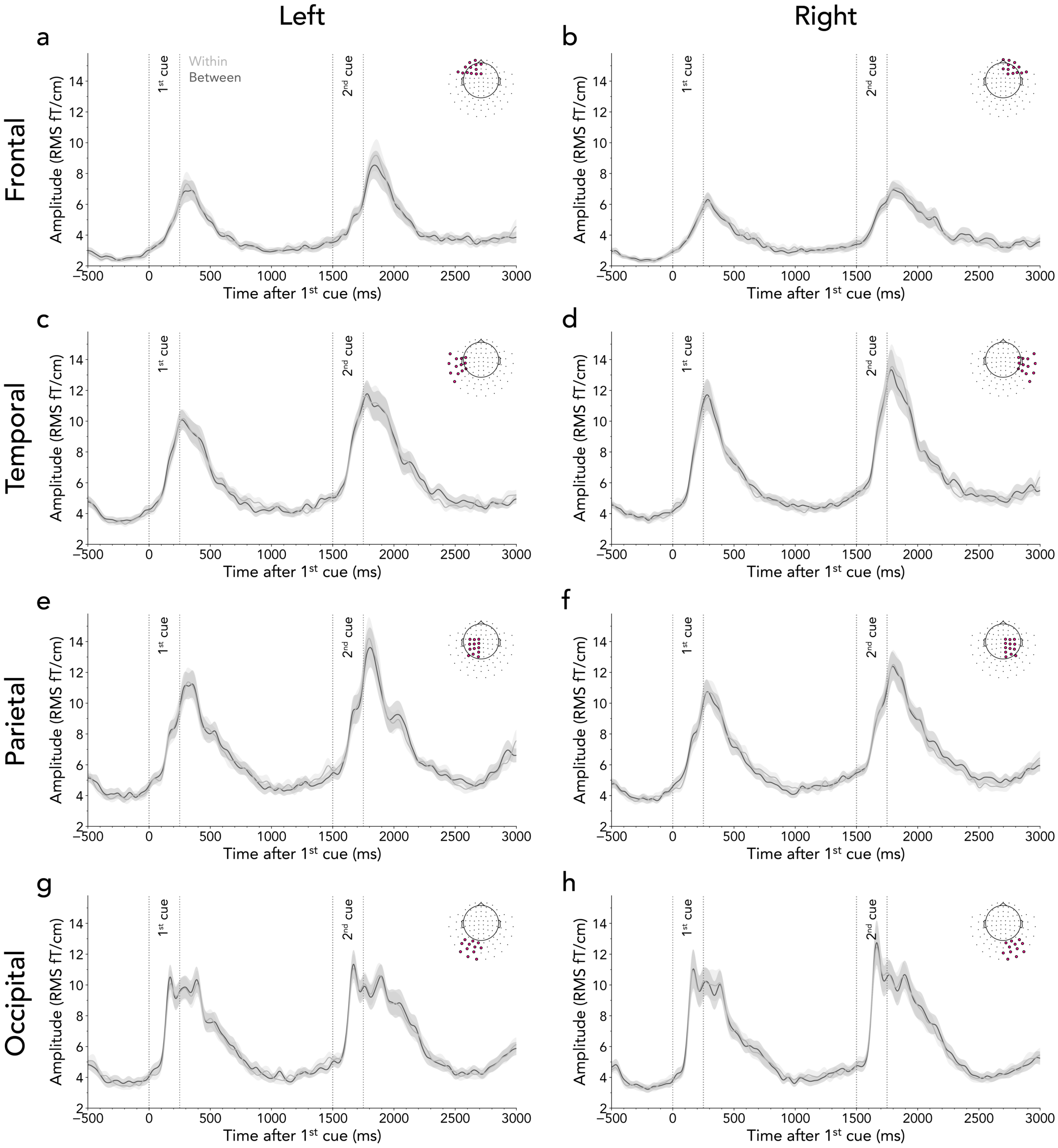

Fig. S1. No differences between shift types in event-related fields (ERFs). (a) ERFs for within- and between-domain shift trials averaged across left frontal sensors (see Appendix SI, Table S1 for sensor locations). Cluster-based non-parametric permutation testing did not reveal any differences between shift types. Time courses show *M* ± *SEM* across participants. **(b)** Same as (a) but for right frontal sensors. **(c)** Same as (a) but for left temporal sensors. **(d)** Same as (a) but for right temporal sensors. **(e)** Same as (a) but for left parietal sensors. **(f)** Same as (a) but for right parietal sensors. **(g)** Same as (a) but for left occipital sensors. **(h)** Same as (a) but for right occipital sensors.

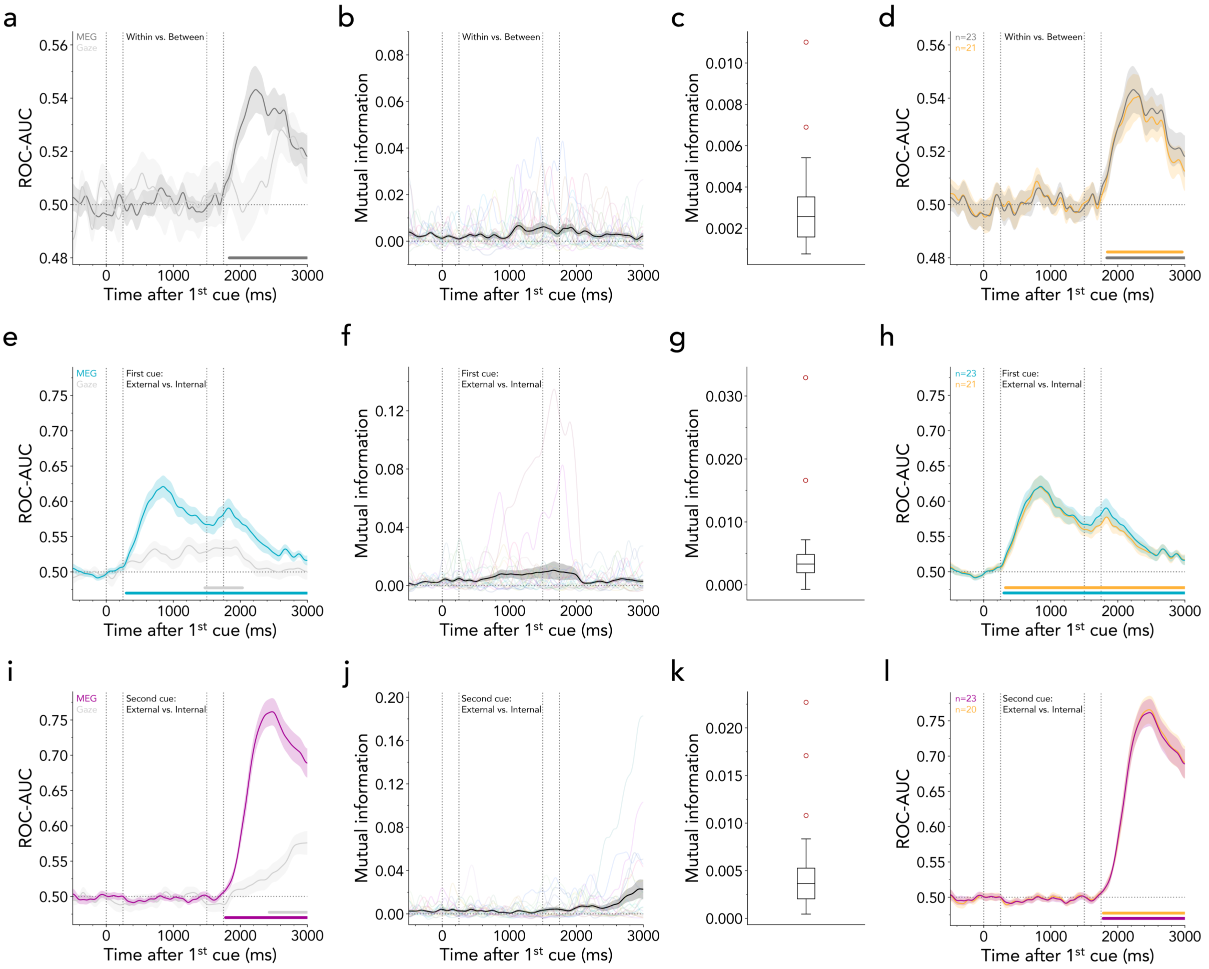

Fig. S2. Eye movements do not account for MEG decoding results. **(a)** Average time-resolved decoding of within- vs. between-domain shifts based on MEG and eye-tracking data. Only MEG data sets were included that had an accompanying eye-tracking data set (*n* = 23). Horizontal lines indicate significant clusters (MEG decoding: ~1840-3000 ms, cluster $p < 0.001$). Time course shows $M \pm SEM$ across participants. **(b)** Average mutual information between MEG and eye-tracking data for the within- vs. between-shift decoding. Faded lines indicate individual participants. **(c)** Box-and-whisker plot according to (33) displaying distribution of average mutual information across -500 to 3000 ms relative to first-cue onset. Red dots represent outliers. **(d)** Average time-resolved MEG decoding of within- vs. between-domain shifts after removing outliers (n=23: ~1840-3000 ms, cluster $p < 0.001$, n=21: ~1840-2960 ms, cluster $p < 0.001$). **(e)** Same as (a) but for external vs. internal first cues (MEG decoding: ~300-3000 ms, cluster $p < 0.001$, eye decoding: ~1470-2030 ms, cluster $p = 0.043$). **(f)** Same as (b) but for external vs. internal first cues. **(g)** Same as (c) but for external vs. internal first cues. **(h)** Same as (d) but for external vs. internal first cues (n=23: ~320-3000 ms, cluster $p < 0.001$, n=21: ~320-3000 ms, cluster $p < 0.001$). **(i)** Same as (a) but for external vs. internal second cues (MEG decoding: ~1780-3000 ms, cluster $p < 0.001$, eye decoding: ~2430-3000 ms, cluster $p = 0.010$). **(j)** Same as (b) but for external vs. internal second cues. **(k)** Same as (c) but for external vs. internal second cues. **(l)** Same as (d) but for external vs. internal second cues (n=23: ~1780-3000 ms, cluster $p < 0.001$, n=20: ~1780-3000 ms, cluster $p < 0.001$).

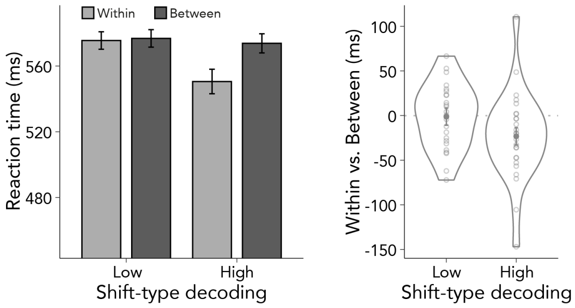

Fig. S3. Within- vs. between-shift decoding is behaviorally relevant. Left panel: Reaction times (RTs) as a function of shift type (within vs. between) and median split (low vs. high). Error bars represent *SEM*. Right panel: Shift cost in RTs defined as the difference of within- and between-shift trials for each half of decoding strength. Dots represent individual participants.

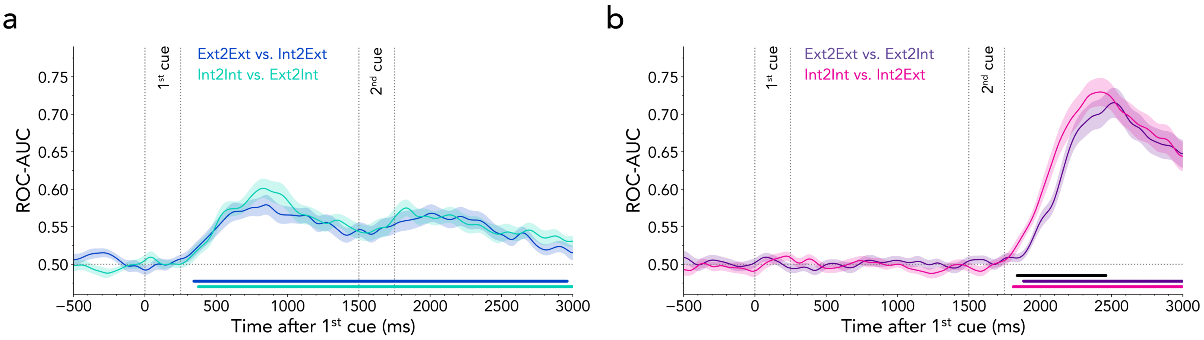

Fig. S4. Time-resolved MEG decoding of different trial types. (a) Average time-resolved classifier performance for distinguishing external-to-external from internal-to-external shifts, and internal-to-internal from external-to-internal shifts. Time courses show *M* ± *SEM* across participants. Colored horizontal lines indicate significant clusters (external-to-external vs. internal-to-external cluster: ~340-2960 ms, cluster $p < 0.001$; internal-to-internal vs. internal-to-external cluster: ~380-3000 ms, cluster $p < 0.001$). **(b)** Same as (a) but decoding internal-to-internal vs. internal-to-external shifts, and external-to-external vs. external-to-internal shifts (external-to-external vs. external-to-internal cluster: ~1880-3000 ms, cluster $p < 0.001$; internal-to-internal vs. internal-to-external cluster: ~1810-3000 ms, cluster $p < 0.001$). Black horizontal line denotes significant difference between decoding time courses (~1840-2460 ms, cluster $p < 0.001$). Since statements regarding the exact onset of an effect are not supported by permutation tests (34), we quantified the timings of the decoding time courses more formally by applying a jack-knife approach to compare the point at which the decoding time courses reached 50% of their peak value. This revealed significantly earlier onset times for internal-to-internal vs. internal-to-external decoding ($M = 2046.240$, $SEM = 20.800$) as compared to external-to-external vs. external-to-internal decoding ($M = 2133.920$, $SEM = 16.738$; $t_{(24)} = -3.379$, $p = 0.002$).

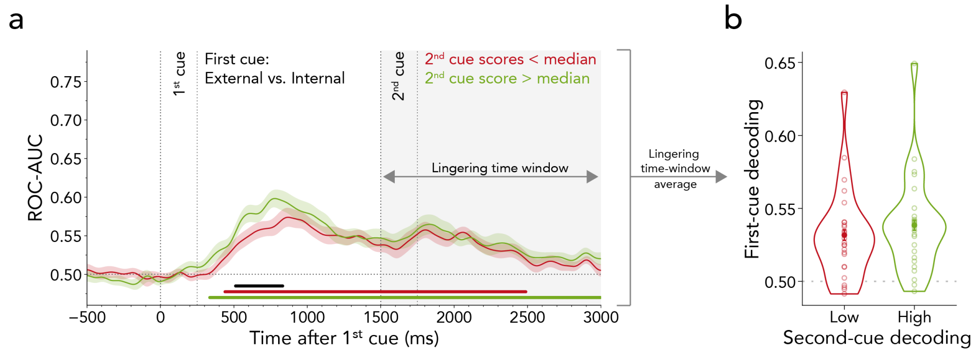

Fig. S5. No evidence for a trade-off in decodability between first- and second-cued attentional domains. (a) We median-split the MEG data into trials with low vs. high decoding performance in distinguishing the second-cued domain following the second cue (i.e., 0-1500 ms after second-cue onset). Next, we trained a classifier separately on both data sets to predict the first-cued domain (external vs. internal). In trials with low and high second-cue decoding, we could successfully predict the first-cued attentional domain after first-cue appearance and the decodability remained significant following the second cue (low decoding: ~440-2490 ms, cluster $p < 0.001$; high decoding: ~340-3000 ms, cluster $p < 0.001$). We did not observe any trade-off in the decoding of the first-cued attentional state between low vs. high second-cue decoding following the second cue (i.e., grey area). There was an earlier difference in the decodability of external vs. internal first-cue trials when the decodability of the second cue was low vs. high (~510-830 ms, cluster $p = 0.012$). Time courses show $M \pm SEM$ across participants. **(b)** Average first-cued domain decoding in the second-cued period (i.e. grey area in (a)) as a function of low vs. high second-cued domain decoding. There was no difference between low and high second-cue decoding in predicting the first-cued domain ($t_{(24)} = 1.348$, $p = 0.190$, $d = 0.270$). Error bars represent *SEM*.

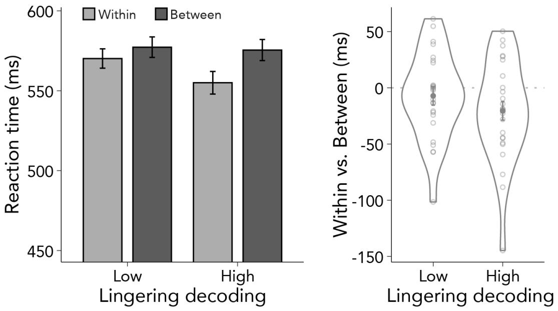

Fig. S6. Lingering first-cue activity is behaviorally relevant. We median-split the MEG data into trials with low vs. high decoding performance in distinguishing the first-cued domain following the second cue (i.e., 0-1500 ms after second-cue onset). Next, we included the median-split factor in our behavioral analysis when comparing within- to between-domain shifts. Left panel: Reaction times (RTs) as a function of shift type (within vs. between) and median split (low vs. high). Error bars represent *SEM*. Right panel: Shift cost in RTs defined as the difference of within- and between-shift trials for each half of decoding strength. Dots represent individual participants.

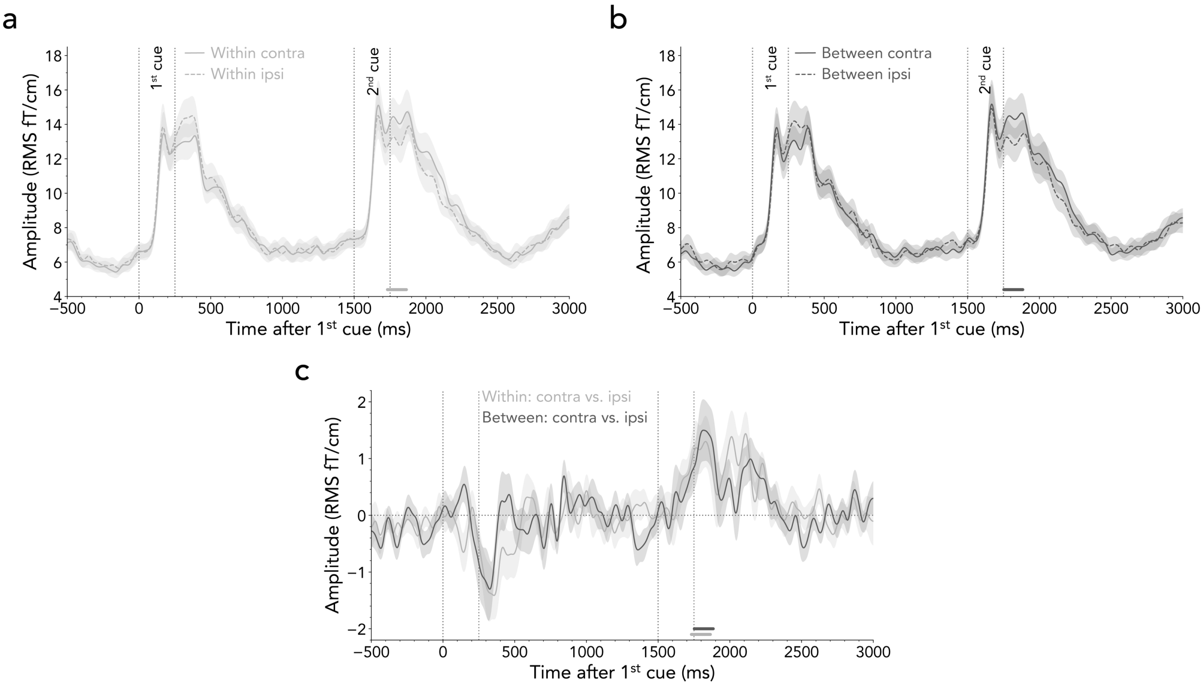

Fig. S7. Lateralized visual activity in posterior regions. (a) Event-related fields (ERFs) for within-domain shift trials from combined posterior gradiometers contra- and ipsilateral to the second-cued hemifield. The dashed and solid line depict the contra- and ipsilateral ERF, respectively. Time courses show $M \pm SEM$ across participants. The difference between contra- vs. ipsilateral lines is the equivalent of the N2pc which is typically observed in electroencephalography studies employing visual-spatial attention tasks. Horizontal lines indicate significant clusters when comparing contra- and ipsilateral ERFs (~1730-1870 ms, cluster p = 0.048). **(b)** Same as (a) but for between-domain shift trials (~1750-1880 ms, cluster p = 0.027). **(c)** Contra- vs. ipsilateral contrast for within- and between-domain shifts. There was no significant difference between conditions.

Table S1. Sensor grouping for event-related fields. For univariate analyses of event-related fields, gradiometer pairs were categorized into eight groups according to different brain regions. Each of the channel groups consists of 12 or 13 sensor pairs.

	Left hemisphere	Right hemisphere
Frontal lobe	MEG0622+0623, MEG0332+0333, MEG0322+0323, MEG0342+0343, MEG0122+0123, MEG0612+0613, MEG0542+0543, MEG0312+0313, MEG0822+0823, MEG0532+0533, MEG0522+0523, MEG0512+0513, MEG0642+0643	MEG1412+1413, MEG1222+1223, MEG1232+1233, MEG1242+1243, MEG1032+1033, MEG1012+1013, MEG1022+1023, MEG0932+0933, MEG1212+1213, MEG0942+0943, MEG0922+0923, MEG0912+0913, MEG0812+0813
Temporal lobe	MEG0222+0223, MEG0212+0213, MEG0132+0133, MEG0232+0233, MEG0242+0243, MEG1542+1543, MEG1512+1513, MEG1622+1623, MEG1612+1613, MEG1522+1523, MEG1532+1533, MEG0112+0113, MEG0142+0143	MEG1312+1313, MEG1322+1323, MEG1442+1443, MEG1432+1433, MEG1342+1343, MEG1332+1333, MEG2612+2613, MEG2412+2413, MEG2422+2423, MEG2642+2643, MEG2632+2633, MEG1422+1423, MEG2622+2623
Parietal lobe	MEG0712+0713, MEG0632+0633, MEG0432+0433, MEG0422+0423, MEG0442+0443, MEG0412+0413, MEG0742+0743, MEG1832+1833, MEG2012+2013, MEG1822+1823, MEG1842+1843, MEG1812+1813, MEG1632+1633	MEG0732+0733, MEG2242+2243, MEG2022+2023, MEG2212+2213, MEG2232+2233, MEG2222+2223, MEG2442+2443, MEG0722+0723, MEG1042+1043, MEG1142+1143, MEG1112+1113, MEG1122+1123, MEG1132+1133
Occipital lobe	MEG2042+2043, MEG1912+1913, MEG1942+1943, MEG1642+1643, MEG1722+1723, MEG1712+1713, MEG1732+1733, MEG1922+1923, MEG2112+2113, MEG1932+1933, MEG1742+1743, MEG2142+2143	MEG2032+2033, MEG2342+2343, MEG2132+2133, MEG2332+2333, MEG2542+2543, MEG2512+2513, MEG2322+2323, MEG2312+2313, MEG2532+2533, MEG2432+2433, MEG2522+2523, MEG2122+2123

SI References

1. D. Gresch, S. E. P. Boettcher, F. van Ede, A. C. Nobre, Shifting attention between perception and working memory. *Cognition* **245**, 105731 (2024).
2. J. Peirce, *et al.*, PsychoPy2: Experiments in behavior made easy. *Behav Res* **51**, 195–203 (2019).
3. Posit team, *RStudio: Integrated Development Environment for R* (Posit Software, PBC, 2022).
4. W. N. Venables, B. D. Ripley, *Modern Applied Statistics with S* (Springer New York, 2002).
5. G. E. P. Box, D. R. Cox, An Analysis of Transformations. *Journal of the Royal Statistical Society: Series B (Methodological)* **26**, 211–243 (1964).
6. A. Gramfort, *et al.*, MEG and EEG data analysis with MNE-Python. *Frontiers in Neuroscience* **7** (2013).
7. A. J. Quinn, M. W. J. van Es, C. Gohil, M. W. Woolrich, OHBA Software Library in Python (OSL). (2022). https://doi.org/10.5281/ZENODO.6875060. Deposited 14 November 2022.
8. S. Taulu, J. Simola, Spatiotemporal signal space separation method for rejecting nearby interference in MEG measurements. *Phys. Med. Biol.* **51**, 1759 (2006).
9. J. van Driel, C. N. L. Olivers, J. J. Fahrenfort, High-pass filtering artifacts in multivariate classification of neural time series data. *Journal of Neuroscience Methods* **352**, 109080 (2021).
10. B. Rosner, Percentage Points for a Generalized ESD Many-Outlier Procedure. *Technometrics* **25**, 165–172 (1983).
11. M. Woolrich, L. Hunt, A. Groves, G. Barnes, MEG beamforming using Bayesian PCA for adaptive data covariance matrix regularization. *Neuroimage* **57**, 1466–1479 (2011).
12. F. Pedregosa, *et al.*, Scikit-learn: Machine Learning in Python. [Preprint] (2018). Available at: http://arxiv.org/abs/1201.0490 [Accessed 24 July 2023].
13. J. E. Hajonides, A. C. Nobre, F. van Ede, M. G. Stokes, Decoding visual colour from scalp electroencephalography measurements. *NeuroImage* **237**, 118030 (2021).
14. T. Grootswagers, S. G. Wardle, T. A. Carlson, Decoding Dynamic Brain Patterns from Evoked Responses: A Tutorial on Multivariate Pattern Analysis Applied to Time Series Neuroimaging Data. *J Cogn Neurosci* **29**, 677–697 (2017).
15. D. J. Hand, R. J. Till, A Simple Generalisation of the Area Under the ROC Curve for Multiple Class Classification Problems. *Machine Learning* **45**, 171–186 (2001).
16. P. Thölke, *et al.*, Class imbalance should not throw you off balance: Choosing the right classifiers and performance metrics for brain decoding with imbalanced data. *NeuroImage* **277**, 120253 (2023).
17. J.-R. King, S. Dehaene, Characterizing the dynamics of mental representations: the temporal generalization method. *Trends Cogn Sci* **18**, 203–210 (2014).
18. D. Kaiser, N. N. Oosterhof, M. V. Peelen, The Neural Dynamics of Attentional Selection in Natural Scenes. *J Neurosci* **36**, 10522–10528 (2016).
19. L. Teichmann, D. Moerel, A. N. Rich, C. I. Baker, The nature of neural object representations during dynamic occlusion. *Cortex* **153**, 66–86 (2022).
20. F. van Ede, S. R. Chekroud, M. G. Stokes, A. C. Nobre, Concurrent visual and motor selection during visual working memory guided action. *Nat Neurosci* **22**, 477–483 (2019).
21. N. Kriegeskorte, R. Goebel, P. Bandettini, Information-based functional brain mapping. *Proc Natl Acad Sci U S A* **103**, 3863–3868 (2006).
22. S. Haufe, *et al.*, On the interpretation of weight vectors of linear models in multivariate neuroimaging. *NeuroImage* **87**, 96–110 (2014).
23. Y. Sun, A. K. C. Wong, M. S. Kamel, Classification of imbalanced data: A review. *International Journal of Pattern Recognition and Artificial Intelligence* **23**, 687–719 (2009).
24. P. Mostert, *et al.*, Eye Movement-Related Confounds in Neural Decoding of Visual Working Memory Representations. *eNeuro* **5** (2018).
25. S. C. Quax, N. Dijkstra, M. J. van Staveren, S. E. Bosch, M. A. J. van Gerven, Eye movements explain decodability during perception and cued attention in MEG. *NeuroImage* **195**, 444–453 (2019).
26. C.-W. Hsu, C.-C. Chang, C.-J. Lin, A Practical Guide to Support Vector Classification. (2003).

27. S. G. Heideman, *et al.*, Anticipatory neural dynamics of spatial-temporal orienting of attention in younger and older adults. *NeuroImage* **178**, 46–56 (2018).
28. S. G. Heideman, F. van Ede, A. C. Nobre, Temporal alignment of anticipatory motor cortical beta lateralisation in hidden visual-motor sequences. *European Journal of Neuroscience* **48**, 2684–2695 (2018).
29. M. K. Abadi, *et al.*, DECAF: MEG-Based Multimodal Database for Decoding Affective Physiological Responses. *IEEE Transactions on Affective Computing* **6**, 209–222 (2015).
30. B. D. van Veen, K. M. Buckley, Beamforming: a versatile approach to spatial filtering. *IEEE ASSP Mag.* **5**, 4–24 (1988).
31. E. Maris, R. Oostenveld, Nonparametric statistical testing of EEG- and MEG-data. *J Neurosci Methods* **164**, 177–190 (2007).
32. J. Miller, T. Patterson, R. Ulrich, Jackknife-based method for measuring LRP onset latency differences. *Psychophysiology* **35**, 99–115 (1998).
33. J. W. Tukey, *Exploratory data analysis* (Addison-Wesley, 1977).
34. J. Sassenhagen, D. Draschkow, Cluster-based permutation tests of MEG/EEG data do not establish significance of effect latency or location. *Psychophysiology* **56**, e13335 (2019).
